# Wiz regulates clustered protocadherin genes by restricting CTCF/cohesin loop extrusion in a genomic-distance biased manner

**DOI:** 10.1371/journal.pgen.1012242

**Published:** 2026-07-16

**Authors:** Tianjie Li, Jingwei Li, Leyang Wang, Haiyan Huang, Qiang Wu

**Affiliations:** 1 Center for Comparative Biomedicine, Key Laboratory of Systems Biomedicine (MOE), Institute of Systems Biomedicine, Shanghai Jiao Tong University, Shanghai, China; 2 Shanghai Key Laboratory of Gene Editing and Cell-based Immunotherapy for Hematological Diseases, State Key Laboratory of Medical Genomics, Ruijin Hospital, Shanghai Jiao Tong University School of Medicine, Shanghai, China; EMBL: European Molecular Biology Laboratory, ITALY

## Abstract

Zinc finger proteins (ZFPs or ZNFs) constitute the largest family of transcription factors in mammals; however, their regulatory mechanism remains largely elusive. Here we propose COP (C2H2-ZFP occupancy predictor), a deep learning-based heuristic screening tool that integrates DNA sequence with protein primary and secondary features to assess ZFP genomic enrichments. Applying COP to the mouse clustered protocadherin (*cPcdh*) gene locus, we identified dozens of C2H2-ZFPs potentially involved in CTCF-mediated gene regulation with Wiz (widely interspaced zinc finger-containing protein) having the highest number of 12 ZFs. We confirmed Wiz enrichments at all of the CTCF-binding site (CBS) elements across the three *Pcdh* clusters by Myc-tagging the endogenous *Wiz* gene. Genetic experiments revealed significant increases of expression levels of the *cPcdh* genes upon *Wiz* deletion in both neuronal cells *in vitro* and in mouse brain *in vivo*. Finally, integrated ChIP-seq, RNA-seq, and 4C-seq analyses demonstrated that Wiz regulates CTCF/cohesin occupancy and long-range enhancer-promoter contacts in a genomic-distance biased manner. Together, these findings reveal a key role for *Wiz* in coupling cohesin occupancy to long-range *cPcdh* regulation and highlight important functions of C2H2-ZFPs in enhancer-promoter interactions.

## Author summary

If you uncoiled the DNA inside a single human cell and stretched it out end-to-end, it would measure about two meters (six feet) long. Yet, this entire blueprint must be packed into the cell’s nucleus, which is microscopic—less than one-tenth the width of a human hair. One of the key mechanisms compacting the three-dimensional (3D) genome is called loop extrusion. In this process, protein complexes such as cohesin act like molecular motors that grab distal DNA elements such as enhancers and promoters together, creating loops of chromatin. Specifically, by squeezing the intervening DNA out into a loop, it physically reels the distant enhancer closer and closer to its target promoter. Once they are brought into direct, physical contact within the loop, the enhancer can successfully activate the promoter, turning on the gene so the cell can produce vital proteins. Using mice as model animals and *cPcdh* as model genes, the authors developed an artificial intelligent program to jointly predict the binding of proteins with DNA elements and found dozens of zinc finger proteins that may participate in CTCF-mediated enhancer-promoter contacts. They then focused on Wiz (widely interspaced zinc finger protein), which possesses the greatest number of zinc fingers. They performed experiments to show the co-localization of Wiz with CTCF or cohesin across both *cPcdh* loci and entire genome and found that Wiz fine-tunes cohesin loop extrusion and *cPcdh* gene expression via regulating long-range enhancer-promoter contacts in a genomic-distance biased manner.

## Introduction

**Fig 1:**
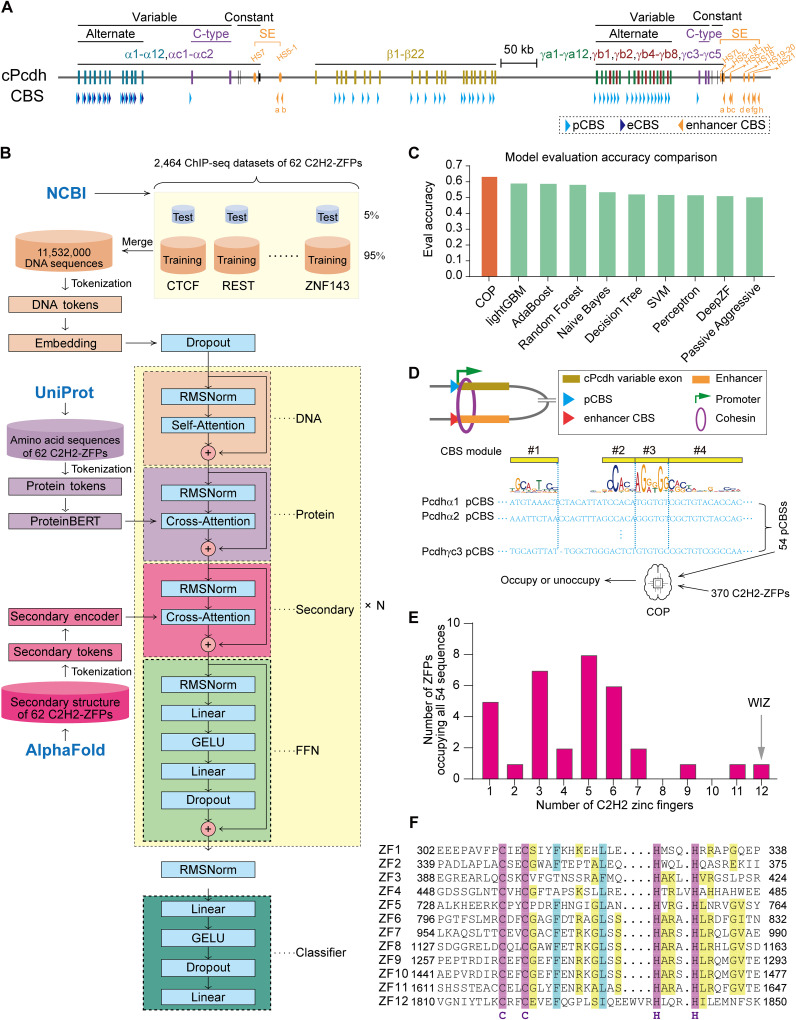
Prediction of C2H2-ZFPs localized at *cPcdh* promoter CTCF sites by COP. **(A)** Genomic organization of the mouse *cPcdh* locus. The mouse *cPcdh* locus comprises three consecutive clusters of *α*, *β*, and *γ*. The *α* and *γ* clusters each contain a variable region of tandem-arrayed alternate and c-type variable exons, and a constant region consisting of three constant exons. Each variable exon can be *cis*-spliced to the downstream set of constant exons, giving rise to 14 *Pcdh*α and 22 *Pcdh*γ isoforms. In contrast, the *β* cluster contains only variable exons and lacks a constant region, encoding 22 *Pcdh*β isoforms. Each variable exon harbors a promoter CBS element (pCBS), with the exceptions of *Pcdh* α*c2*, β*1*, γ*c4*, and γ*c5*. Moreover, each variable exon of the *Pcdh*α cluster harbors an exonic CBS element (eCBS). The *Pcdh* α super-enhancer (*HS7* and *HS5-1)* and *Pcdh* βγ super-enhancer (*HS7L*, *HS5-1aL*, *HS5-1bL*, and *HS18-21*) are highlighted in orange. CBS, CTCF binding site; eCBS, exonic CBS; HS, hyper sensitive site; kb, kilobases; pCBS, promoter CBS; Pcdh, protocadherin; SE, super-enhancer. **(B)** Schematic of the COP model. DNA embeddings are first processed by a self-attention layer to capture intra-sequence dependencies. They then undergo two sequential cross-attention steps, first with protein primary sequence embeddings, followed by secondary structure embeddings, to integrate protein features. The fused representations are refined by a feedforward network (FFN). This entire process is repeated N times to enable hierarchical feature learning. Finally, the resulting output is fed into a classifier. COP, C2H2-ZFP occupancy predictor; FFN, feedforward network; GELU, Gaussian error linear unit; RMSNorm, root mean square layer normalization. **(C)** Benchmark comparison with existing models. The COP prediction accuracy (orange) outperforms that of other approaches. **(D)** Illustration of COP prediction of ZFPs that cooperate with CTCF at enhancer-promoter (E-P) looping hubs of the *cPcdh* locus, using 54 promoter CBS (pCBS) elements and 370 C2H2-ZFPs as input. The top schematic depicts a CTCF-anchored E-P loop at the *Pcdh* clusters. CTCF facilitates gene activation by bringing distal enhancers into the proximity of promoters via stabilizing cohesin-mediated loop extrusion at forward-oriented pCBS and reverse-oriented enhancer CBS elements. **(E)** COP predicts Wiz as a top candidate with the highest number of 12 ZFs. **(F)** Amino acid sequence alignment of the twelve C2H2 ZFs of the mouse Wiz. The conserved cysteine (C) and histidine (H) residues that coordinate the zinc ion are highlighted in magenta.

The *cPcdh* locus encodes dozens of cadherin-like cell-adhesion proteins via the mechanism of stochastic and monoallelic promoter choice combined with alternative splicing [1–9]. The mouse *cPcdh* locus spans nearly 1 Mb and comprises three consecutive clusters of *α*, *β*, and *γ*. Both the *Pcdh* α and *γ* clusters are organized into a 5’ variable region with a tandem array of large exons and a 3’ constant region that contains a single set of common exons (Fig 1A). Each 5’ large variable exon encodes a signal peptide, followed by six cadherin ectodomains, the transmembrane domain, and a membrane-proximal cytoplasmic region. The 3’ shared constant exons encode a membrane-distal C-terminal cytoplasmic region. Each 5’ variable exon is spliced to the respective set of downstream 3’ constant exons, generating 14 *Pcdh*α isoforms (12 alternate and 2 c-type) and 22 *Pcdh*γ isoforms (12 a-type, 7 b-type, and 3 c-type). The *Pcdh*β cluster contains only 22 variable exons each encoding a *Pcdh*β isoform [1,10]. Each variable exon, except for *Pcdh* α*c2*, β*1*, γ*c4*, and γ*c5*, contains a promoter CTCF-binding site (pCBS) element (Fig 1A). These pCBS elements are paired with CBS within a downstream distal super-enhancer to form CTCF/cohesin-mediated enhancer-promoter (E-P) chromatin loops which determine stochastic isoform choice [7,10–13]. Each alternate variable exon of the *Pcdh*α cluster also contains an exonic CBS (eCBS) element that reinforces the chromatin loop via a “double clamping” mechanism using two pairs of CBS elements with opposite orientations [12]. This pairing is achieved via CTCF anchoring or stabilizing cohesin “loop extrusion” at convergent CBS elements within variable promoters and super-enhancers [5,8,9,12,13].

The eleven zinc finger (ZF) domains of CCCTC-binding factor (CTCF) play a key role in antiparallel recognition of diverse *cPcdh* CBS elements for cohesin directional “loop extrusion” [5,7,8,14–16]. Recently, several other ZF proteins (ZFPs) have been implicated in regulating the *cPcdh* gene expression via directional recognition of diverse DNA elements, such as the repressor element-1 silencing transcription factor (REST) or neuron-restrictive silencer factor (NRSF) [17], ZNF143 [18], and ZNF274 [19]. They utilize different combinations of ZF domains, each typically interacting with a DNA triplet to bind to a variety of regulatory elements in a sequence-specific manner. In mammals, there are approximately ~800 C2H2-ZFPs that constitute the largest family of DNA-binding transcription factors [20,21]. Most ZFPs contain clustered ZF domains, with a great variety of residues both within and between ZFs, which posing a major challenge to accurately define their DNA-binding specificity. Based on ZFP ChIP-seq (Chromatin immunoprecipitation followed by high-throughput sequencing) and ChIP-exo (ChIP with lambda exonuclease digestion) *in vivo* as well as *in vitro* binding datasets, several computational models have been developed for predicting their binding sites [21–25]. For example, DeepZF mainly utilizes zinc finger polypeptide and target sequences as inputs to predict its DNA binding [26]. However, the biological function of ZFP enrichments at genomic sites remains challenging owing to both their flexible direct recognition of DNA motifs and indirect interacting with co-occupied proteins or even RNAs.

The widely interspaced zinc finger-containing protein (Wiz), which contains 12 dispersed ZF domains (ZF1–12), functions as a regulator of CTCF/cohesin-mediated chromatin loops [27]. Wiz was originally identified in the mouse brain as two isoforms: a long ZF1–11 and a short ZF6–11 [28]. The association of Wiz with the G9a/GLP histone methyltransferase complex suggests a transcriptional repression function [29,30]. Recently, its repressive role in fetal hemoglobin (HbF) gene expression was uncovered through a chemical screen, revealing *Wiz* as a promising therapeutic target for sickle cell diseases (SCD) [31]. Finally, Wiz has been implicated in transcriptional insulation and is essential for maintaining embryonic stem cell identity [32]. Here we developed a C2H2-ZFP occupancy predictor (COP) using attention mechanisms to jointly model the relevance of ZFP amino acid sequences, secondary structures, and target DNA sequences. COP inferred that Wiz has the potential to bind all of the pCBS elements within the *Pcdh* gene clusters. By deleting *Wiz* in the neuroblastoma Neuro-2a (N2a) cells *in vitro* and in mouse brain *in vivo*, combined with integrated RNA-seq, ChIP-seq, and 4C-seq analyses, we found that Wiz impairs CTCF binding, restricts chromatin cohesin, and suppresses *cPcdh* gene expression in a genomic-distance biased manner.

## Results

### COP screening of C2H2-ZFPs localized at the *cPcdh* promoter CBS elements

To assess potential C2H2-ZFP localization at specific DNA sequences in a systematic manner, we developed a deep neural network model named COP, which comprises separate encoding modules for DNA sequences, protein sequences, and protein secondary structures (Fig 1B). DNA sequences are tokenized and embedded with rotary positional encoding and processed by self-attention layers. Protein sequences are embedded with a pre-trained ProteinBERT model (S1A Fig), whereas protein secondary structures are encoded using a dedicated transformer encoder (S1B Fig). Two cross-attention layers are applied to integrate protein sequence and secondary structural information into the DNA sequence representation. The model parameters are optimized to classify protein-DNA pairs as occupied or unoccupied.

We trained COP on 62 mouse C2H2-ZFPs with 2,464 ChIP-seq datasets (S1 Table). For each protein, we curated 3,000 high-confidence occupied sites, yielding a dataset of 186,000 positive (occupied) protein-DNA pairs. To construct negative controls, we combined each DNA sequence with non-occupying proteins, applying gradient-based sampling to select challenging negative pairs with high prediction loss in early epochs, yielding a dataset of 11,346,000 negative (unoccupied) protein-DNA pairs. This strategy generated a total of 11,532,000 protein-DNA pairs for the training set. Benchmark evaluations demonstrated that COP achieved higher accuracy (63.6%) compared to LightGBM (59.4%) [33], AdaBoost (59.2%) [34], and other known models [26,35–39] (Fig 1C). These results show that COP outperforms traditional machine learning models in terms of prediction accuracy.

To screen for C2H2-ZFPs involved in CTCF-mediated regulation of *cPcdh* gene expression, we applied COP to each of the 54 pCBS elements from the *cPcdh* promoters to identify localized C2H2-ZFPs (Fig 1D). Amino acid sequences and secondary structures were collected for 370 C2H2-ZFPs from UniProt [40] and AlphaFoldDB [41], respectively. These datasets then input together with pCBS sequences into COP to infer whether individual C2H2-ZFPs occupy pCBS elements of the *Pcdh* clusters. This revealed 34 C2H2-ZFPs (Figs 1E and S1C–S1L), with 12 trained on COP and 22 untrained (S2 Fig), enriched at the 54 pCBS elements. Among the 34 C2H2-ZFPs, Wiz contains the highest number of 12 ZFs (Figs 1E, 1F and S1L). Wiz is of particular interest regarding *cPcdh* regulation owing to its high expression levels in the brain [28].

### Wiz colocalizes with CTCF at CBS elements of *cPcdh* promoters and enhancers

**Fig 2:**
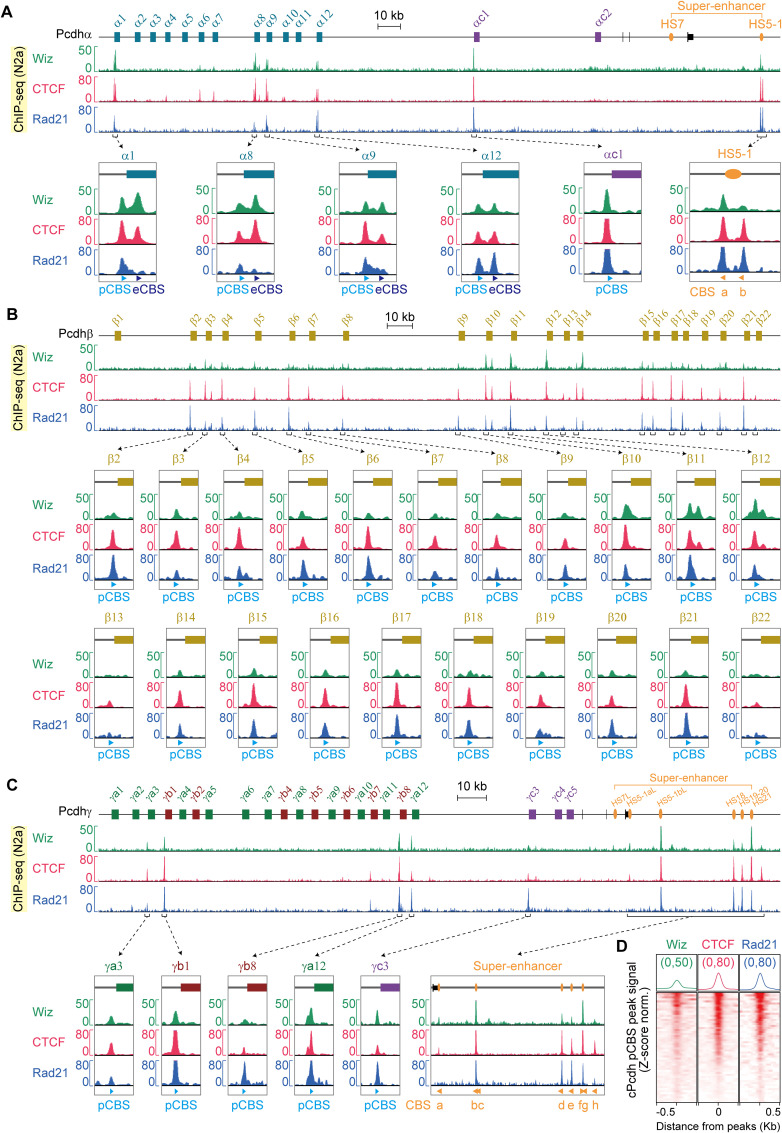
Wiz colocalizes with CTCF and cohesin at both promoter and enhancer regions of the *cPcdh* locus in N2a cells. **(A-C)** ChIP-seq profiles of Wiz, CTCF, and Rad21 at the *Pcdh* α (**A**), *β* **(B)**, and *γ* (**C**) clusters in N2a single-cell clone expressing endogenous Wiz with a C-terminal Myc tag. The pCBS and eCBS elements within individual genes as well as enhancer CBS elements are highlighted in close-up views. **(D)** Aggregated ChIP-seq signals showing Wiz, CTCF, and Rad21 enrichments at the promoters of the *cPcdh* locus in N2a cells. ChIP-seq signals were normalized using RPKM (reads per kilobase per million mapped reads). Data represent merged ChIP-seq signals from three replicates.

To evaluate occupancy of Wiz at the *cPcdh* gene complex, we generated stable cell clones with Myc-tagged endogenous *Wiz* via CRISPR/Cas9-mediated DNA-fragment editing and single-cell screening in N2a cells (S3A Fig) [9,42]. We then performed ChIP-seq experiments with a specific antibody against Myc to map Wiz enrichments and found that Wiz is colocalized with CTCF and cohesin at the c*Pcdh* promoter and super-enhancer regions (Fig 2). In the *Pcdh*α cluster, Wiz is enriched at both pCBS and eCBS elements of *Pcdh* α*1*, α*8*, α*9*, α*12*, and α*c1* as well as two CBS elements (*a* and *b*) flanking the *HS5-1* super-enhancer (Fig 2A). In the *Pcdh*β cluster, Wiz is enriched at all pCBS elements (Fig 2B). In the *Pcdh*γ cluster, Wiz is enriched at the pCBS elements of *Pcdh* γ*a3*, γ*b1*, γ*b8*, γ*a12*, and γ*c3* as well as eight CBS elements of *a-h* within the downstream super-enhancer (Fig 2C). The enrichment of Wiz and CTCF at the *cPcdh* promoters (Fig 2D) aligns well with the previously reported localization of Wiz at active promoters and CBS elements in the adult mouse cerebellum [43].

**Fig 3:**
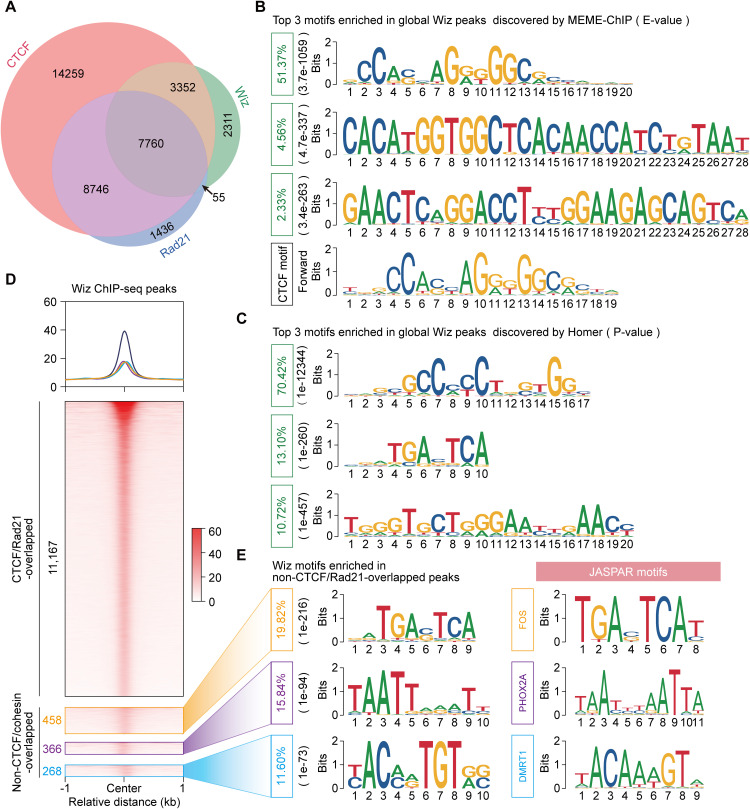
Wiz colocalizes with CTCF and cohesin genome wide in N2a cells. **(A)** Venn diagram of Wiz, CTCF, and Rad21 ChIP-seq peaks in N2a single-cell clone, showing genome-wide colocalization of Wiz with CTCF and cohesin. **(B** and **C)** Motif discovery for global Wiz peaks by both MEME-ChIP (**B**) and HOMER **(C)**, showing prevalence of CTCF consensus motif [13]. **(D)** Heatmap of Wiz ChIP-seq showing Wiz peaks overlapped and non-overlapped with CTCF/Rad21. The non-overlapping Wiz peaks were grouped based on the types of motif shown in **(E)**. **(E)** Motifs enriched at Wiz peaks non-overlapped with CTCF/Rad21. *De novo* motif discovery by MEME-ChIP identified three predominant motifs, with similarity to FOS (MA0476.2), PHOX2A (MA0713.1), and DMRT1 (MA1603.2) binding sites in JASPAR.

Genome-wide comparison of our ChIP-seq data on Wiz, CTCF, and Rad21 (a subunit of the cohesin complex) revealed that 82.4% of Wiz peaks (11,112 of 13,478) overlapped with CBS elements (Fig 3A). Among these, 69.8% (7,760 of 11,112) were jointly occupied by both CTCF and Rad21 (Fig 3A), suggesting a close association of Wiz with CTCF/cohesin. Consistently, both MEME-ChIP and Homer identified the CBS elements as the prominent motif at Wiz peaks (Fig 3B and 3C). Further motif discovery revealed three predominant motifs at Wiz peaks lacking CTCF or Rad21 occupancy, highly similar to the consensus sequences of the FOS, PHOX2A (paired-like homeobox 2A), and DMRT1 (doublesex- and mab-3-related transcription factor 1) binding sites in the JASPAR database (Fig 3D and 3E). These three motifs are not identified by the previous Wiz ChIP-seq in the mouse adult cerebellum, suggesting a cell-specific role of Wiz in FOS, PHOX2A, or DMRT1 mediated transcriptional regulation.

### Wiz ablation leads to increased *cPcdh* gene expressions *in vitro*

**Fig 4:**
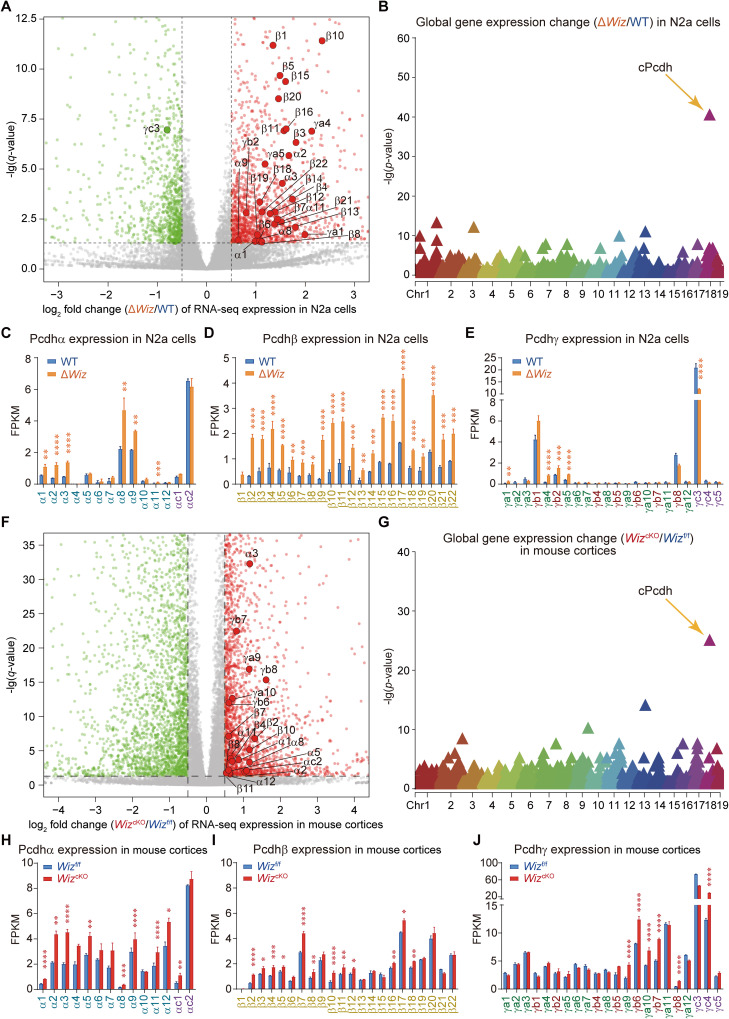
Loss of *Wiz* increases *cPcdh* expression levels in both neuronal N2a cells and mouse cortices. **(A)** Volcano plot depicting differentially expressed genes in neuronal N2a cells upon *Wiz* knockout. Red and green dots represent up-regulated (log_2_ fold change (FC) > 0.5, *p* < 0.05) and down-regulated genes (log_2_ FC < -0.5, *p* < 0.05), respectively, in Δ*Wiz* N2a single-cell clones compared to the wild-type (WT) control clone. **(B)** Manhattan plot depicting the localized enrichment of differentially expressed genes (1-Mb sliding window) in Δ*Wiz* versus WT N2a cell clones, with *cPcdh* being the top-ranked. Each point represents a single 1-Mb genomic bin, and the *p*‑value indicates the statistical significance of spatial enrichment of differentially expressed genes for that interval. **(C-E)** RNA-seq showing expression levels of members of the *Pcdh* α (**C**), *β* (**D**), and *γ* (**E**) gene clusters in Δ*Wiz* compared to WT N2a cell clones. **(F)** Volcano plot depicting differentially expressed genes in P0 mouse cortices upon conditional knockout of *Wiz*. Red and green dots represent up-regulated (log_2_ FC > 0.5, *p* < 0.05) and down-regulated (log_2_ FC < -0.5, *p* < 0.05) genes, respectively, in *Wiz* conditional knockout (*Wiz*^cKO^) versus the *Wiz*^f/f^ control mice. **(G)** Manhattan plot showing the strongest enrichment of upregulated genes (1-Mb sliding window) at the c*Pcdh* locus in P0 cortices from *Wiz*^cKO^ mice versus *Wiz*^f/f^ controls. **(H-J)** RNA-seq showing expression levels of members of the *Pcdh* α (**H**), *β* (**I**), and *γ* (**J**) gene clusters in *Wiz*^cKO^ mice compared to *Wiz*^f/f^ controls, showing a significant increase in c*Pcdh* expression levels upon *Wiz* knockout in the cerebral cortices. RNA-seq was performed in duplicate for each sample. For cortical samples, two individual mice of each genotype (*Wiz*^f/f^ and *Wiz*^cKO^) were used. For Δ*Wiz* N2a cells, data from two independent clones were merged. For *Wiz*^f/f^ or *Wiz*^cKO^ mice, data from two independent animals were merged. FPKM, fragments per kilobase of exon per million reads mapped. Data as mean ± standard deviation (SD); unpaired Student’s *t*-test. *p ≤ 0.05, **p ≤ 0.01, ***p ≤ 0.001, ****p ≤ 0.0001; detailed *p*-values are provided in S3 and S4 Tables.

We employed CRISPR/Cas9-mediated DNA fragment editing to delete the entire *Wiz* coding region in N2a cells and obtained two homozygous *Wiz*-knockout (Δ*Wiz*) single-cell clones (S3B Fig) [9,42]. Western blotting and RNA-seq confirmed that both Wiz mRNA and protein were depleted in Δ*Wiz* N2a cell clones (S4A–S4C Fig). Genome-wide analyses revealed 4,187 differentially expressed genes upon *Wiz* deletion, comprising 2,786 upregulated and 1,401 downregulated genes (Figs 4A and S4D) (S2 Table). Of these, 9.5% of upregulated genes and 19.6% of downregulated genes harbor Wiz peaks within ±2 kb of their transcriptional starting sites (TSS) (S4E Fig).

Remarkably, 1.15% (32 *cPcdh* genes) of the upregulated genes are members of the *Pcdh* clusters, resulting in a unique, ~ 32-fold enrichment for dysregulated transcripts at this gene complex in comparison with the rest of the genome (Poisson test, *P* = 5.07 × 10^-41^, as determined by a 1-Mb sliding window applied to n = 4,187 transcripts) (Fig 4B). Specifically, members of the *Pcdh*α (Fig 4C), *Pcdh*β (Fig 4D), and *Pcdh*γ (Fig 4E) clusters are upregulated as shown in heatmap (S4F Fig). In particular, every member of the *Pcdh*β cluster is upregulated upon *Wiz* deletion (Figs 4D, S4F and S4G), conspicuously similar to the *Pcdh*β upregulation upon *Wapl* conditional knockout or knockdown [7,9]. Finally, ChIP-seq experiments revealed significant increases of active histone marks of H3K4me3 and H3K27ac at the promoter and super-enhancer regions of the *Pcdh* clusters upon *Wiz* deletion (S5 Fig), consistent with *cPcdh* gene upregulation. Collectively, *Wiz* deletion leads to activation of the *cPcdh* genes, suggesting that Wiz acts as a repressor for these genes in N2a cells.

### *Wiz* deletion in the brain leads to increased *cPcdh* gene expressions *in vivo*

We next generated *Wiz* conditional knockout mice to investigate its role in the brain *in vivo* because constitutional homozygous null allele of *Wiz* leads to embryonic lethality at early development stages [44]. We first established a *Wiz*-floxed mouse strain (*Wiz*^*+/f*^) using CRISPR/Cas9-mediated homologous recombination via pronuclear microinjection (S6A Fig). Genotyping of 28 P0 chimeric founders identified ten mice carrying correctly targeted *Wiz*-floxed alleles. These chimeric mice were crossed with WT C57BL/6J to establish a germline-transmitted *Wiz*^*+/f*^ line. Specifically, four heterozygous *Wiz*^*+/f*^ mice with two simultaneous *loxP* insertions in a single allele were obtained (S6B Fig) and subsequently crossed with *Emx1*-*Cre* (Empty spiracles homeobox 1-Cre) transgenic mice to generate *Wiz*^*+/f*^;*Emx1*-*Cre* progeny. Further crossing of these mice with *Wiz*^*f/f*^ animals yielded *Wiz*^*f/f*^;*Emx1*-*Cre* line, i.e., cortex-specific *Wiz* knockouts (*Wiz-*cKO).

Conditional knockout of *Wiz* via Cre-mediated recombination resulted in depletion of both Wiz transcript and protein in postnatal day 0 (P0) mouse cortices, as examined by RNA-seq and Western blotting, respectively (S7A–S7C Fig). RNA-seq analyses uncovered widespread transcriptional alterations *in vivo* in the cerebral cortices of *Wiz*-cKO mice compared with control littermates. At P0, a total of 7,607 genes were differentially expressed upon *Wiz* deletion (3,407 upregulated vs. 4,200 downregulated) (Figs 4F and S7D) (S3 Table). In particular, 0.79% (27 *cPcdh* genes) of the upregulated genes are mapped within the *cPcdh* locus, far exceeding their expected frequency. Specifically, a ~ 15-fold enrichment was observed relative to the genomic background (Poisson test, *P* = 1.71 × 10^-25^), based on a 1-Mb sliding-window analysis across the 7,607 dysregulated transcripts (Fig 4G). Members of the *Pcdh* α (Fig 4H), *β* (Fig 4I), and *γ* (Fig 4J) clusters are upregulated as shown in heatmap (S7E Fig) and fold changes (S7F Fig). Together, both *in-vitro* and *in-vivo* deletions of *Wiz* lead to activation of the *cPcdh* genes, suggesting a repressive role of Wiz in regulating these clusters.

### Wiz represses *cPcdh* by restricting CTCF/cohesin enrichments at promoters and enhancers

**Fig 5:**
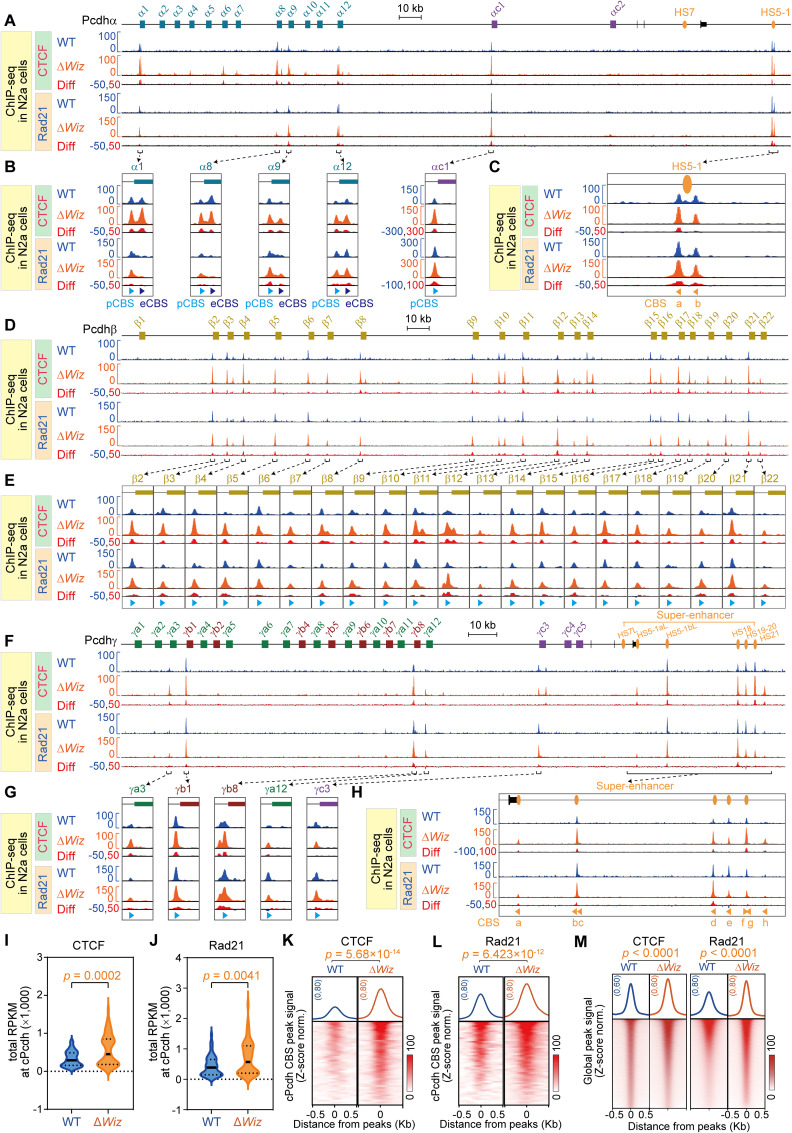
Increased enrichments of CTCF and Rad21 at the *cPcdh* CBS elements upon *Wiz* knockout in N2a cells. **(A-H)** ChIP-seq profiles of CTCF and Rad21 at the *Pcdh* α **(A-C)**, *β* **(D** and **E)**, or *γ* (**F-H**) gene cluster. **(I** and **J)** ChIP-seq quantifications showing a significant increase of CTCF (**I**) and Rad21 (**J**) enrichments in N2a cells upon *Wiz* deletion. **(K** and **L)** Aggregated peak analyses showing a significant increase of CTCF (**K**) and Rad21 (**L**) enrichments at the *cPcdh* locus in N2a cells upon *Wiz* deletion. **(M)** Heatmaps of ChIP-seq signals across the genome in ∆*Wiz* compared to WT cells, showing a global increase of CTCF and Rad21 enrichments upon *Wiz* deletion. ChIP-seq signals were normalized using RPKM (reads per kilobase per million mapped reads). For WT N2a cells, CTCF and Rad21 signals were merged from two and four replicates, respectively. For Δ*Wiz* N2a cells, data from two independent deletion clones were combined, each with two (CTCF) or four (Rad21) replicates.

We performed ChIP-seq experiments to assess CTCF/cohesin enrichments upon *Wiz* deletion and found a significant increase in CTCF and Rad21 enrichments at the *cPcdh* locus (Fig 5). Specifically, in the *Pcdh*α cluster, CTCF and cohesin enrichments markedly increased at both pCBS and eCBS elements of *Pcdh* α*1*, α*8*, α*9*, α*12*, and α*c1* as well as at the two CBS elements in the *HS5-1* super-enhancer region (Fig 5A–5C). In addition, there is a remarkable increase of CTCF and cohesin enrichments at the pCBS element of each member of the *Pcdh*β cluster (Fig 5D and 5E). Moreover, there is a similar increase in the *Pcdh*γ cluster at both variable promoters and downstream super-enhancer (Fig 5F–5H). Finally, total reads and aggregated peak analyses showed a significant increases of both CTCF (Fig 5I and 5K) and cohesin (Fig 5J and 5L) enrichments within the *cPcdh* gene complex upon *Wiz* deletion. A similar genome-wide increase of CTCF and cohesin enrichments was observed upon *Wiz* deletion (Fig 5M). In summary, these data suggest that *Wiz* represses *cPcdh* gene expression by restricting CTCF/cohesin enrichments at the CBS elements of promoters and super-enhancers.

### Wiz regulates *cPcdh* gene expression in a genomic-distance biased manner

**Fig 6:**
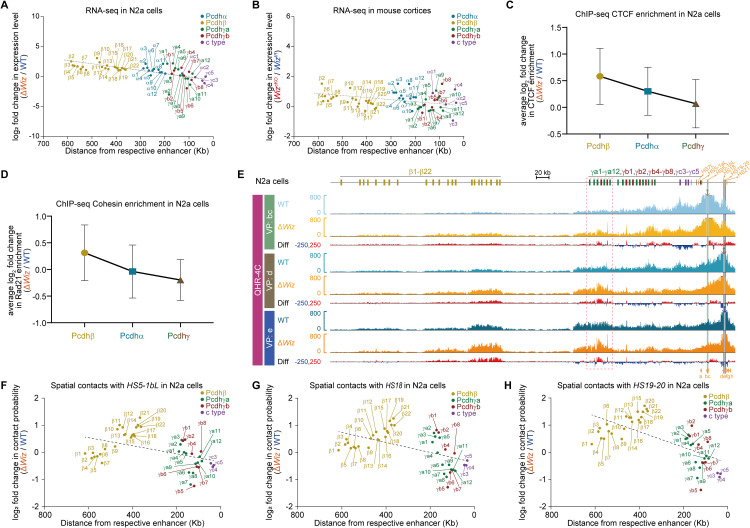
Wiz ablation increases *cPcdh* expression in a genomic-distance biased manner. **(A)** Scatter plot depicting expression changes of *cPcdh* genes in Δ*Wiz* versus wild-type (WT) N2a cell clones *in vitro*, plotted against their linear genomic distances from respective enhancer. Linear regression: R^2^ = 0.4497, *p* < 0.0001. **(B)** Scatter plot depicting expression changes of *cPcdh* genes in P0 cortices from Wiz conditional knockout (*Wiz*^cKO^) versus *Wiz*^f/f^ mice *in vivo*, plotted against their linear genomic distances from respective enhancer. Linear regression: R² = 0.0117, *p* = 0.4226. **(C** and **D)** Average log_2_ fold changes of CTCF (**C**) and Rad21 (**D**) enrichments at pCBS elements within the *Pcdh* β, *α*, and *γ* clusters in N2a cells upon *Wiz* deletion. **(E)** 4C profiles using *CBSbc*, *CBSd*, or *CBSe* as a viewpoint (VP) in ∆*Wiz* compared to WT N2a cells, showing increased chromatin contacts with the distal *Pcdh*βγ genes upon *Wiz* deletion. Differences (∆*Wiz* versus WT) are shown below the 4C profiles. **(F-H)** Scatter plot showing a genomic-distance biased increase in contact probabilities of *Pcdh*βγ genes with *HS5-1bL* **(F)**, *HS18* **(G)**, or *HS19-20* (**H**) enhancer within the super-enhancer in N2a cells upon *Wiz* deletion. Linear regression: *HS5-1bL*, R^2^ = 0.2124, *p* = 0.0016; *HS18*, R^2^ = 0.3034, *p* = 0.0001; *HS19-20*, R^2^ = 0.4611, *p* < 0.0001. For Δ*Wiz* N2a cells, data from two independent clones were merged. For *Wiz*^cKO^ mice, data from two independent animals were merged.

Given the emerging role of cohesin-mediated loop extrusion in controlling *cPcdh* transcription via long-range enhancer-promoter communication [4,5,7,11–13,45], we next examined whether the transcriptional activation of *cPcdh* genes upon *Wiz* loss exhibits linear genomic-distance bias. Indeed, analyses of RNA-seq data revealed a pronounced genomic-distance biased increase in *cPcdh* expression levels in N2a cells *in vitro* (Fig 6A) upon *Wiz* deletion, with enhancer-distal members displaying stronger upregulation than proximal ones. Consistent with this, bulk RNA-seq data from mouse cortices *in vivo* also showed a positive correlation between genomic distance from the enhancer and the magnitude of expression change upon Wiz deletion (Fig 6B). Although this trend did not reach statistical significance, likely due to the substantial cellular heterogeneity of cortices, it reinforces the notion that *Wiz* restricts *cPcdh* gene expression in a genomic-distance biased manner.

To investigate the underlying mechanism, we quantified CTCF and Rad21 ChIP-seq datasets and found a genomic-distance biased increase of CTCF/cohesin occupancy at *cPcdh* genes upon *Wiz* deletion (Fig 6C and 6D), suggesting that *Wiz* may stall cohesin loop extrusion. We then performed a series of QHR-4C (Quantitative high-resolution chromosome conformation capture copy) experiments using the element of *CBSbc*, *CBSd*, or *CBSe* within the *Pcdh*βγ super-enhancer region as a viewpoint and found a significant increase of long-distance chromatin interactions with *Pcdh*β and distal members of the *Pcdh*γ cluster (Fig 6E). Quantitative 4C analyses revealed a genomic-distance biased increase of spatial contacts between diverse variable promoters and downstream super-enhancers (Fig 6F–6H). Together, these data suggest that *Wiz* restricts or inhibits cohesin loop extrusion from super-enhancer to tandem promoters.

## Discussion

The *cPcdh* gene complex encodes an enormous diversity of cadherin-like cell adhesion proteins functioning as a sophisticated “molecular barcode” system essential for establishing the precise architecture of the mammalian brain [1,4,6–8,46–49]. These cell-surface adhesion molecules undergo stochastic and combinatorial expression, providing each neuron with a unique tag of biochemical identity for self/non-self discrimination [50–55]. This molecular diversity is also the foundation for neuronal self-avoidance, a process where sister dendritic branches from the same neuron recognize one another via homophilic Pcdh interactions and subsequently repel apposite membranes, ensuring the neuron maximally covers its territory without self-entanglement [56–60]. Beyond self/non-self discrimination and self-avoidance, cPcdh proteins also mediate synaptic pruning and the refinement of complex neural circuits [6,61]. When this molecular barcode is disrupted — often due to aberrant 3D genome organization or epigenetic dysregulation — the resulting “miswiring” of the brain can lead to neurodevelopmental disorders such as depression, autism, and schizophrenia [62,63]. The unique variable and constant genome architecture and great differential distances of tandem variable promoters to the downstream distal super-enhancers impose intrinsic biases on promoter choice and isoform selection [1,4,5,7,13]. Fine tuning cohesin processivity along the linear chromatin and balanced spatial contacts of variable promoters with distal super-enhancers via topological chromatin insulators are key to overcome the intrinsic genomic-distance biases [5,8,9].

Diverse C2H2-type ZFPs constitute the largest family of transcription factors in the mammalian genome [20,21]. Each ZF consists of a conserved ββα fold stabilized by a single zinc ion coordinated by two cysteines of β-sheets and two histidines of an α-helix. Although the DNA recognition code is incompletely understood, each ZF domain recognizes a DNA triplet via four specific residues of an α-helix located on the opposite side of the zinc ion. The true power of this family lies in its modular versatility. By linking multiple ZFs in tandem as a daisy chain, these proteins can recognize long, specific DNA elements with diverse affinities, as seen for the 11-finger protein CTCF in 3D genome architecture. We hypothesized that members of the ZFP family may collaborate with CTCF to fine tune the delicate expression patterns of *cPcdh* genes and proposed an AI-based model of COP to identify CTCF-colocalizing C2H2-ZFPs, inferring 34 C2H2-ZFPs potentially enriched at the repertoire of the 54 *cPcdh* pCBS elements. Among them, the forkhead box (FOX) family members of FOXP1/2 [64], the GLI family members of GLI2/3 [65], the ZIC family members of ZIC2–5 [66], as well as POGZ (Pogo transposable element derived with ZNF domain) [67], have been reported to be important for neural development or associated with neurodevelopmental disorders. Further evaluation of their regulatory role in neuronal transcription of *cPcdhs* would uncover the hidden complexity of *cPcdh* gene regulation in neural circuit assembly. Among the 34 C2H2-ZFPs, *Wiz* harbors the highest number of 12 ZFs which are widely interspaced. Its ZFs may contribute to CBS enrichments via protein-protein interactions or RNA binding rather than direct DNA binding. For example, Wiz C-terminal ZF is essential for interacting with the G9a/GLP complex [29,30]. RNA binding was recently shown to be prevalent among C2H2-ZFPs [68–71]. In a UV crosslinking and immunoprecipitation (CLIP) experiment, 148 of 150 analyzed C2H2-ZFPs were observed to bind directly to RNA in human cells [71].

Wiz was originally identified by a homologous screening [28] and later was shown to maintain H3K9me1/2-marked heterochromatin by stabilizing the G9a/GLP histone methyltransferase complex on chromatin [29,30]. Wiz-haploinsufficiency results in a reduced level of the *Pcdh*β gene expression, suggesting that Wiz functions as a transcriptional activator [43]. However, homozygous *Wiz* deletion showed prominent increases of the *Pcdh*β genes in cultured N2a cells *in vitro* and in mouse brain *in vivo* (Fig 4). This inconsistency may result from the different sizes of the deleted *Wiz* regions. Our *Wiz* cKO mice targeted the complete set of six C-terminal ZFs (S6 Fig), while previous *Wiz* N-Ethyl-N-nitrosourea (ENU) mutagenesis causes frameshifts of only the two C-terminal ZFs [43,44]. In addition, differences in genetic background between C57BL/6 (in particular an transposon element insertion in one *Wiz* exon) and FVB/NJ mouse strains could also contribute to the discrepant effects on the *cPcdh* gene expression [43,72]. Nevertheless, our *Wiz* cKO mice may have an anxiety-like phenotype, as reported for Wiz-haploinsufficiency mice [43], as they were frequently observed hiding in cage corners and adopting a curled or hunched posture. Finally, integrated and quantitative analyses suggest that Wiz regulates *cPcdh* gene expression by restricting CTCF/cohesin loop extrusion from distal super-enhancers to diverse variable promoters in a genomic-distance biased manner (Figs 5 and 6), explaining the previous observation that distal enhancers preferentially regulate distal *cPcdh* genes whereas proximal enhancers tend to contact with close promoters [5,45].

Wiz is both a transcription factor and a structural regulator of chromatin loops [30,32,43,73]. Since Wiz colocalizes and interacts with CTCF and cohesin complex [31,32,73], Wiz may stabilize G9a/GLP H3K9 methylation enzymes on chromatin regions containing CBS elements via its C-terminal ZF [29,30,73] and recruit the CtBP corepressor complex via its three PXDLS-like motifs to silence genes [30]. However, we observed only a slight decrease in H3K9 mono- and dimethylation within the promoter regions of the *cPcdh* locus upon *Wiz* deletion (S8 and S9A–S9C Figs). In addition, global H3K9 methylation levels showed no significant alterations at transcriptional start sites (TSSs) following *Wiz* deletion (S9D–S9F Fig). By profiling genome-wide distribution of ChIP-seq peak summits of Wiz, CTCF, and Rad21 at their co-occupied sites, we found that Wiz peak summits were located between those of CTCF and Rad21 (S9G Fig). Remarkably, upon Wiz depletion, Rad21 peaks shifted ~3 bp toward CTCF sites (S9H Fig), suggesting that Wiz fine-tunes cohesin extrusion activity (S9I Fig), similar to the role of ZNF143 in relative CTCF/cohesin positioning [18]. By colocalizing with CTCF at insulators, promoters, and enhancers, Wiz regulates their spatial interactions [31,32]. The high conservation of human and mouse Wiz polypeptides (84% identity) and the dispersed organization of 12 ZFs with long linkers are consistent with its architectural or structural role in chromatin looping [27,32,43], similar to the structural role at pericentric heterochromatin region of the dispersed ZF protein ZFP512 [74]. Wiz represses *cPcdh* gene expression not by “heterochromatinization” of the locus via H3K9 methylation but by stalling cohesin processivity or loop extrusion, affecting predominantly members of the *Pcdh*β cluster owing to its great distance from the downstream distal super-enhancer. This could explain the more pronounced increases of spatial contacts of super-enhancers with distal promoters than with proximal promoters, providing a mechanistic explanation for the preferential activation of distal *cPcdh* genes upon *Wiz* deletion.

Collectively, these observations point to a previously unrecognized architectural role of Wiz in modulating loop extrusion and chromatin topology. By restricting longer-range enhancer-promoter contacts across the three *Pcdh* clusters via constraining cohesin chromatin loop extrusion, Wiz regulates *cPcdh* expression in a genomic-distance biased manner.

## Materials and methods

### Ethics statement

All experiments were approved by the Institutional Animal Care and Use Committee (IACUC) of Shanghai Jiao Tong University (protocol#: 1602029).

### Animals

All mouse strains were maintained at 23 °C on a 12/12 h light-dark cycle (7:00–19:00) in an SPF (specific pathogen free) facility.

### Plasmid construction

Plasmids used for expressing sgRNA were constructed as previously described [42,75]. In brief, each pair of complementary oligonucleotides (S4 Table) was annealed in NEBuffer 2 (NEB, B7002S) to produce a double-stranded DNA (dsDNA) fragment with 5’ overhangs of ‘ACCG’ and ‘AAAC’ at two ends, respectively. Each annealed dsDNA fragment was cloned into the *Bsa*I site of pGL3 vector under the U6 promoter.

For generation of single-cell clones with *Wiz* endogenously Myc-tagged at the C-terminus, a donor plasmid was designed for Cas9-mediated homologous recombination. Left and right homologous arms were amplified from the mouse genomic DNA by PCR using a pair of primers (S4 Table) and cloned into the *Eco*RI and *Xba*I sites of the pTNT vector (Promega, L5610). The Myc-tag coding sequence was inserted between the left and right homologous arms by overlapping PCR using primers containing the Myc-tag coding sequence (S4 Table).

For generation of *Wiz*-floxed mice, two donor plasmids were designed for targeting the fourth and the tenth introns of *Wiz*, respectively. For each donor, the left and right arms were amplified by PCR from the mouse genomic DNA, respectively, with the reverse primer of the left arm and the forward primer of the right arm containing complementary *loxP* sequences. The amplified products of left and right homology arms were assembled into the linearized pUC19 vector using the Clone Express MultiS OneStep Cloning Kit, to generate the final donor plasmid with the *loxP* sequence flanked by the two homologous arms. All plasmids were confirmed by Sanger sequencing.

### Cell culture

Mouse Neuro-2a (N2a) cells were cultured in modified Eagle’s medium (Gibco, 11095080) containing 10% fetal bovine serum (Sigma-Aldrich, F0193), 1% penicillin–streptomycin (Gibco, 15140122), and 1% non-essential amino acids (Gibco, 11140050). Cells were maintained at 37 °C in a humidified environment with 5% CO_2_.

Cells were passaged when reaching approximately 80% confluence. After removal of the medium, cells were gently washed once with 1 ml of 1 × PBS (Gibco, 70011044). Detachment was achieved by adding 1 ml of 0.25% trypsin–EDTA (Gibco, 25200056) and incubating the cells at 37 °C for 3 min. Enzymatic digestion was terminated by the addition of 1 ml of complete medium. The cell suspension was transferred to a 15-ml conical tube and centrifuged at 900 rpm for 5 min at room temperature. Following centrifugation, the supernatant was removed and the cells were resuspended in 1 ml of fresh medium. For subculturing, 250 µl of the resulting cell suspension was seeded into a new dish containing 5 ml of complete medium.

### Generation of single-cell clone with *Wiz* endogenously Myc-tagged

To tag Myc at the C-terminus of endogenous Wiz, sgRNA and donor plasmids were co-transfected with the Cas9-expression plasmid into the N2a cells, using Lipofectamine 3000 (Invitrogen, L3000015) according to the manufacturer’s protocol. Transfected cells were cultured for 2 days, followed by selection with 2 μg/ml puromycin for four days. Cells were subsequently transferred to the puromycin-free medium and maintained for one week for recovery. After recovery, the transfected population cells were genotyped by PCR to first confirm the CRISPR insertion. Specifically, genomic DNA was extracted and used as a template for PCR amplification with specific primers (S4 Table) to detect the targeted insertion of Myc-tag sequence. Cell populations with targeted insertion were then subjected to single-cell clone screening. Briefly, the transfected cells were diluted and seeded into the 96-well plates at approximately one cell per well. A total of 254 single-cell clones were screened, and one homozygous single-cell clone was obtained for Myc-tagging at the C-terminus of endogenous Wiz. All single-cell clones were genotyped by Sanger sequencing.

### Generation of Δ*Wiz* single-cell clones

To knock out *Wiz*, a pair of sgRNAs was designed to target the first and the tenth introns of *Wiz* that span the coding sequence of ZF1–12. The two sgRNA-expression plasmids were co-transfected with the Cas9-expression plasmid into the N2a cells, using Lipofectamine 3000. Transfected cells were cultured, puromycin-selected, and subjected to single-cell clone screening as described above. Genomic DNA was extracted and PCR-amplified using specific primers (S4 Table) to detect the targeted deletion of *Wiz*. A total of 128 clones were screened and two homozygous Δ*Wiz* single-cell clones were obtained. All clones were genotyped by Sanger sequencing.

### Synthesis of sgRNA and Cas9 mRNA for microinjection

Both sgRNA and Cas9 mRNA were synthesized via *in-vitro* transcription. For sgRNA transcription, a DNA template containing a T7 promoter followed by sgRNA targeting sequence and scaffold sequence was PCR-amplified from the pGL3 plasmid with a pair of primers (S4 Table). The amplified template was purified with the QIAquick PCR Purification kit (Qiagen, 28104), extracted with phenol-chloroform, precipitated with ethanol, and resuspended in RNase-free water. The sgRNA was transcribed using the MEGAshortscript T7 kit (Invitrogen, AM1354) and purified with the MEGAclear kit (Invitrogen, AM1908) according to the manufacturer’s protocol. The transcribed sgRNA was eluted in 30 μl of Elution buffer, quantified with a NanoDrop 2000 Spectrophotometer, and aliquoted at 2.5 μg per tube for storage at -80 °C to avoid repeated freeze-thaw cycles.

For Cas9 mRNA *in-vitro* synthesis, the pcDNA3.1-Cas9 vector, containing Cas9 coding sequence under a T7 promoter, was linearized with *Xba*I (NEB, R0145S), purified by gel extraction, extracted with phenol-chloroform, and precipitated by ethanol. After ethanol precipitation, the plasmid DNA pellet was resuspended in 15 μl of RNase-free water and used for *in-vitro* transcription with the mMACHINE T7 ULTRA kit (Invitrogen, AM1345). The transcribed Cas9 mRNA was then subjected to DNase digestion, poly(A) tailing, and purification with the MEGAclear kit. Purified Cas9 mRNA was quantified by NanoDrop 2000 Spectrophotometer and aliquoted at 5 μg per tube for storage at -80 °C.

### Generation of *Wiz*-floxed mice for conditional knockout of *Wiz*

C57BL/6J female mice of 3–6 weeks old were induced for superovulation by subjecting to an intraperitoneal injection of 10 U of pregnant mare serum gonadotropin (PMSG) (Solarbio, P9970) and 48h-later, a second injection of 10 U of human chorionic gonadotropin (hCG) (MCE, HY-107953). After the injection of hCG, each female mouse was placed in an independent cage with a stud male of C57/BL6J for crossing. Plugged females were euthanized, and fertilized zygotes were collected from oviducts and cultured in M2 medium at 37 °C with 5% CO_2_ for microinjection.

To construct the floxed *Wiz* allele, two donor DNA templates were co-injected with Cas9 mRNA and dual single-guide RNAs (sgRNAs) targeting intronic regions flanking the *Wiz* exons 6–13 (S6A Fig). Each donor carried a single *loxP* site, enabling precise insertion of one *loxP* site at flanking intronic regions. Two sgRNAs were designed to target the introns 5 and 13 of *Wiz*, respectively, to induce Cas9-mediated homologous recombination. Specifically, 1.25 μg each of the two sgRNAs was mixed with 5 μg each of two donor plasmids and 5 μg of Cas9 mRNA in RNase-free water to prepare a 50-μl injection mixture. For microinjection, chambers containing M2 medium drops equilibrated under mineral oil were prepared in advance. Approximately 2 picolitre of the injection mixture was injected into the pronucleus of each zygote. After recovery for 30 min, morphologically normal zygotes were transferred into the oviducts of pseudopregnant ICR females.

### Mouse genotyping

The chimeric F0 mice were genotyped by PCR using specific primers (S4 Table). In brief, a small piece of the mouse tail was snipped into an Eppendorf tube. The tail tissue was lysed in 30 μl of the Solution A (25 mM NaOH) at 95 °C for 20 min and neutralized with 30 μl of the Solution B (25 mM Tris-HCl pH 6.8). The 2 μl of the neutralized tail lysis solution was used as a template to screen for the targeted mutations by polymerase chain reaction (PCR under the conditions: 95 °C, 3 min; 95 °C, 15 s, 58 °C, 15 s, 72 °C, 15 s for 40 cycles; and a final extension at 72 °C, 5 min) using specific primers. PCR products were Sanger-sequenced for genotyping. The chimeric F0 mice with desired insertions were crossed with wildtype C57BL/6J mice to generate heterozygous (*Wiz*^*f/+*^) F1 mice. The targeted F1 male and female mice were crossed to generate the F2 mice. The homozygous (*Wiz*^*f/f*^) F2 mice were genotyped by Sanger sequencing and used for generation of *Wiz* conditional knockout.

### Western blot

Total proteins were extracted from harvested cultured cells and P0 mouse cortices. For the preparation of cortex samples, whole brains were rapidly dissected from P0 mice, freed from skin and cartilage, and immersed in ice-cold NeuroBasal medium (Gibco, 21103049). The cerebral cortices were isolated, mechanically minced into small pieces using fine forceps, and digested with 0.25% Trypsin-EDTA at 37 °C for 15 min. The enzymatic digestion was terminated by adding PBS containing 10% fetal bovine serum (FBS). Subsequently, both the dissociated cortical cells and the harvested cultured cells were washed twice with ice-cold 1 × PBS and lysed in pre-chilled RIPA lysis buffer (Beyotime, P0013B) supplemented with protease inhibitors. The samples were then subjected to ultrasonic homogenization (2 sec pulse, 5 sec interval, 2 cycles). After incubation on ice for 30 min, the lysates were centrifuged at 12,000 g for 15 min at 4 °C, and the supernatant was collected for subsequent analysis or stored at -80 °C. Protein concentrations were quantified using a BCA Protein Assay Kit (Beyotime, P0012). The samples were then mixed with an equal volume of 2 × SDS loading buffer, denatured at 95 °C for 10 min, and centrifuged briefly. Equal amounts of proteins were loaded onto SDS-PAGE gels and separated at 80 V for 20 min, followed by 120 V for 1.5 h. Subsequently, proteins were transferred onto nitrocellulose (NC) membranes (Cyvita, 10600002) using a semi-dry transfer system. The transfer assembly was constructed with filter papers and membranes pre-saturated in the transfer buffer, ensuring the removal of all air bubbles. The transfer process was conducted at a constant voltage of 105V for 2.5 h. Following transfer, the membranes were blocked with 5% non-fat milk at room temperature for 1 h and washed twice with 1 × PBS. The membranes were then incubated with Wiz (Abcam, ab92334) and β-actin (Abmart, T40104) antibodies overnight at 4 °C. After washing three times with 1 × PBS, the membranes were incubated with appropriate secondary antibodies diluted in 5% non-fat milk for 1 h at room temperature. Finally, the protein bands were visualized and scanned using the Odyssey Infrared Imaging System (LI-COR Biosciences), and the integrated optical density of the bands was analyzed for quantification.

### RNA-seq

RNA-seq experiments were performed as previously described with minor modiﬁcations [13]. Briefly, cultured cells or dissociated cortical cells were lysed in 1 ml of TRIzol (Invitrogen, 15596026) by vortexing vigorously for 15 min at room temperature. The sample was then centrifuged at 12,000 g for 10 min at 4 °C. The supernatant was transferred to a new microcentrifuge tube, and 0.2 ml of chloroform was added. After vigorous shaking for 15 s, the sample was incubated for 3 min at room temperature. After centrifugation at 12,000 g for 15 min at 4 °C, the aqueous phase was carefully transferred to a new RNase-free tube and mixed with 0.5 ml of isopropanol to precipitate RNA. After a 10-min incubation at room temperature, RNA was precipitated by centrifugation at 12,000 g for 10 min at 4 °C. The precipitated RNA was washed once with 1 ml of 75% ethanol, air-dried for 5 min, and resuspended in 30 μl of nuclease-free water. The concentration of RNA was measured using a spectrophotometer (NanoDrop, 2000). The high-quality RNA, with an A260/A280 ratio of ~2.0, was subjected to library preparation.

RNA-seq libraries were prepared using the Universal V6 RNA-seq Library Prep kit for Illumina (Vazyme, NR604–01) following the manufacturer’s instructions. Briefly, the polyadenylated mRNA was isolated using oligo(dT) coupled to magnetic beads (Vazyme, N401) from 100 ng of total RNA and fragmented by heating at 94 °C for 8 min. The first cDNA strand was synthesized by reverse transcription with a random primer (N6). After synthesizing the second strand of DNA, the adapter was ligated. The ligated DNA was cleaned by AMPure XP Beads (Beckman, A63881), and mixed with 5 μl of the P5 primer, 5 μl of the P7 primer, and 25 μl of HiFi Amplification Mix (Vazyme, NR604) for PCR-amplification (98 °C, 30 s; 98 °C, 10 s, 60 °C, 30 s, 72 °C, 30 s for 10 cycles; and a final extension at 72 °C, 5 min). RNA-seq libraries were sequenced on an Illumina platform. RNA-seq experiments were performed in duplicate for each cell sample from wild-type (WT) and two independent Δ*Wiz* clones. For cortical samples, RNA‑seq experiments were performed for two individual mice of *Wiz*^f/f^ or *Wiz* cKO.

### ChIP-seq

Cultured cells were first washed twice with PBS, detached by trypsin digestion, resuspended in 10 ml of medium to neutralize the trypsin digestion. Formaldehyde (Thermo, 28908) was added to a final concentration of 1% for cross-linking at room temperature for 10 min. The glycine was added to a final concentration of 125 mM and incubated at room temperature for 5 min to quench the cross-linking reactions. Cross-linked cells were centrifuged at 2,500 g for 10 min at 4 °C and washed with ice-cold PBS. Washed cells were lysed on ice using 1 ml of ChIP Lysis Buffer (10 mM Tris-HCl pH 7.5, 1 mM EDTA, 1% Triton X-100, 0.1% sodium deoxycholate, 150 mM NaCl, 1 × protease inhibitors) for 30 min, and centrifuged at 2,500 g for 5 min at 4 °C to obtain cell nuclei.

The isolated nuclei were resuspended in 0.7 ml of ChIP Lysis Buffer and sonicated using a Bioruptor Plus sonicator (Diagenode) in a non-contact mode at high power at a train of 30 cycles of 30 s ON/30 s OFF to yield 200–1,000 bp DNA fragments. The sonicated samples were centrifuged at 14,000 g for 10 min at 4 °C. The supernatants were transferred to a new tube and precleared with 50 μl of protein A/G-agarose beads (Millipore, 16–157) at 4 °C with slow rotation for 2 h. After centrifugation at 2,000 g for 1 min at 4 °C, the protein A/G-agarose beads were discarded, and the supernatants were transferred to a new tube. The primary antibody was added and incubated overnight at 4 °C with slow rotation for immunoprecipitation. 50 μl of the protein A/G-agarose beads were added and incubated at 4 °C with slow rotation for 3 h. The samples were centrifuged at 2,000 g for 1 min at 4 °C and followed by sequential washes with Low Salt Washing Buffer (0.1% SDS, 1% Triton X-100, 2 mM EDTA, 20 mM Tris-HCl pH 8.0, 150 mM NaCl), High Salt Washing Buffer (0.1% SDS, 1% Triton X-100, 2 mM EDTA, 20 mM Tris-HCl pH 8.0, 500 mM NaCl), LiCl Washing Buffer (0.25 M LiCl, 1% NP-40, 1% sodium deoxycholate, 1 mM EDTA, 10 mM Tris-HCl pH 8.0), and TE Buffer (10 mM Tris pH 8.0, 1 mM EDTA).

The washed antibody/protein/DNA complexes were eluted twice with 100 μl of Elution Buffer (50 mM Tris-HCl pH 8.0, 10 mM EDTA, 1% SDS) by incubation at 65 °C for 30 min with vortexes. The 200-μl eluted solution was mixed with 200 μl of TE buffer, de-cross-linked at 65 °C overnight with vortexes, and sequentially digested with 2 μl of 10 mg/ml RNase A at 37 °C for 2 h and 8 μl of 10 mg/ml proteinase K at 55 °C for 2 h. The DNA was puriﬁed with 400 μl of phenol/chloroform, precipitated, and resuspended in 20 μl of nuclease-free water. DNA concentration was measured by PicoGreen reagents. A total of 10 ng of DNA was used as a template for library construction using NGS Ultima Pro DNA Library Prep Kit (Yeasen, 12201ES96). The generated ChIP-seq libraries (S5 Table) were sequenced on an Illumina platform.

### QHR-4C

QHR-4C experiments were carried out as previously described with minor modifications [5]. Briefly, about 1 × 10^7^ cells were harvested as described above and crosslinked with formaldehyde at a final concentration of 2% at room temperature for 10 min. Crosslinking was quenched by adding 2 M glycine to a final concentration of 200 mM. Crosslinked cells were centrifuged at 220 g for 5 min, washed with 10 ml of ice-cold PBS, and centrifuged again at 220 g for 5 min. Cells were then permeabilized twice with 200 μl of ice-cold 4C permeabilization buffer (50 mM Tris-HCl pH 7.5, 150 mM NaCl, 5 mM EDTA, 0.5% NP-40, 1% Triton X-100, and 1 × protease inhibitors) each for 10 min. After centrifugation, the pellet was resuspended in 73 μl of water, 10 μl of 10 × *Dpn*II buffer, and 2.5 μl of 10% SDS. The resuspended cells were incubated at 37 °C for 1 h with shaking at 900 rpm. 12.5 μl of 20% Triton X-100 was added into the reaction to quench SDS and incubated at 37 °C for 1 h with shaking at 900 rpm. The cells were then digested *in situ* overnight at 37 °C with 2 μl of 10 U/μl *Dpn* II while shaking at 900 rpm. After the inactivation of *Dpn* II at 65 °C for 20 min, the pellets of the nuclei were collected by centrifuging at 1,000 g for 1 min, and the supernatant was removed completely, which ensures that the subsequent ligation reaction can be performed in a small volume. Proximity ligation was carried out for 24 h at 16 °C with 1 μl of T4 DNA ligase (400 U/μl) in 100 μl of 1 × T4 ligation buffer. The ligated product was then de-cross-linked by heating at 65 °C for 4 h in the presence of 1 μl of proteinase K (10 mg/ml) to digest proteins. The DNA was then extracted using phenol-chloroform. One μl of glycogen (20 mg/ml) was added to facilitate DNA precipitation. The precipitated DNA was resuspended in 50 μl of water and fragmented to an average size of 200–500 bp using a Bioruptor sonicator (low-power setting, 30 s ON and 30 s OFF, 12 cycles).

The fragmented DNA was used as the template for linear amplification using a 5’ biotinylated primer (S4 Table) complementary to the viewpoint fragment. The amplification was performed in a 100-μl PCR reaction (95 °C, 2 min; 95 °C, 15 s, 58 °C, 25 s, 72 °C, 90 s for 82 cycles; and a final extension at 72 °C, 5 min). The PCR products were denatured at 95 °C for 5 min and immediately chilled on ice to generate single-stranded DNA (ssDNA). The generated ssDNA was then enriched and purified with Streptavidin Magnetic Beads (Thermo), followed by ligation with annealed adaptors in a 45-μl ligation reaction (20 μl of DNA-coated beads, 4.5 μl of 10 × T4 ligation buffer, 10 μl of 30% PEG 8000, 1 μl of 50 μM adaptor, 0.9 μl of T4 DNA ligase, 8.6 μl of water). After ligation, the beads were washed twice with 1 × Binding and Washing Buffer (5 mM Tris-HCl pH 7.5, 1 M NaCl, 0.5 mM EDTA) to remove unligated adaptors, and finally resuspended in 10 μl of water. Using the bead-bound DNA as template, the 4C library was PCR-amplified using high-fidelity DNA polymerase (Vazyme, P505-d1) with specific primers (S4 Table) under the cycling condition (95 °C, 3 min; 95 °C, 15 s, 60 °C, 30 s, 72 °C, 1 min for 19 cycles; and a final extension at 72 °C, 5 min). The amplified 4C library was purified with High Pure PCR Product Purification Kit (Roche) and sequenced on an Illumina platform. 4C experiments were performed in duplicate for each sample.

### Artificial intelligence (AI) model

#### Processing of mouse C2H2-ZFP data.

All amino acid sequences of mouse C2H2-ZFPs were retrieved from UniProt [40] using the query “(ft_zn_fing: C2H2) AND (organism_id: 10090)”, yielding 380 entries. Predicted 3D protein structure models were obtained from the AlphaFold Protein Structure Database (AlphaFoldDB) [41]. Of these 380 proteins, six lacked corresponding structural models in AlphaFoldDB, and four exhibited discrepancies in sequence length between UniProt and AlphaFoldDB entries. These ten proteins were therefore excluded. Consequently, high-confidence structural and sequence data for 370 mouse C2H2 ZFPs (S1 Table) were retained for downstream analyses.

The 3D protein structures were converted into secondary structure assignments using DSSP [76], which classifies residues into nine canonical secondary structure types: *α*-helix, residue in isolated *β*-bridge, extended strand participating in *β-*ladder, 310-helix, *π*-helix, *κ*-helix (poly-proline II helix), hydrogen-bonded turn, bend, and none (unstructured). Two functionally important motifs, namely, C2H2-ZF motif, essential for sequence-specific DNA binding, and the KRAB domain, commonly found adjacent to C2H2-ZF arrays, were explicitly annotated as distinct secondary structure classes to preserve their biological relevance in downstream analyses.

#### Processing of public ChIP-seq data.

Among the 370 mouse C2H2-ZFPs, ChIP-seq data were available for 62 in the NCBI SRA (S1 Table). Raw sequencing reads were downloaded and aligned to the mm9 reference genome using Bowtie2 (v2.3.4.1). Peak calling was performed with MACS (v1.4.2), and peaks overlapping with the ENCODE mm9 blacklist [77] were excluded. To consolidate nearby binding sites, peaks with at least 50-bp of overlap were clustered. Within each cluster, a single representative peak was retained, especially, the one located at the 90th percentile of *p*-values, to minimize redundancy and suppress spurious peak calling. To ensure uniformity in peak width, the top 10% widest and narrowest peaks were discarded. To further reduce false positives, the top 10% of peaks with the highest *p*-values (least significant) and lowest *p*-values (potentially artifactual) were also excluded. Finally, to balance statistical robustness and computational efficiency, 3,000 peaks were randomly sampled per protein, and 100-bp sequences centered on each peak summit were extracted for downstream analyses.

#### Tokenization of input sequences.

Input DNA sequences were tokenized using a vocabulary comprising the characters: m, c, A, C, G, T, and N, corresponding to the indices 0–6, respectively. Specifically, m denotes a padding token used for sequence length masking. The character c serves as a sequence classification token, positioned at the beginning of each sequence, and functions as the input to the classification head for downstream prediction, following the BERT-style [CLS] token paradigm [78]. A, C, G, and T represent the four canonical DNA nucleotides. N represents an ambiguous or unknown nucleotide. For each pCBS element within the *cPcdh* genes, the 38-bp core motif was extended symmetrically to a total length of 256 bp prior to input into COP. This extension was guided by transcription factor binding peak data from the ReMap database [79], aiming to maximize coverage of bound C2H2-ZFPs.

Input protein sequences were encoded using ProteinBERT [80] with a vocabulary of 26 tokens (indexed 0–25). To ensure uniform sequence length, a padding token p was introduced. Sequence boundaries were explicitly marked by adding a start token s at the beginning and an end token e at the terminus. The tokens A, C, D, E, F, G, H, I, K, L, M, N, P, Q, R, S, T, V, W, and Y represent the 20 standard amino acids. The remaining tokens, U, X, and o, correspond to selenocysteine, undefined amino acids, and non-canonical amino acids, respectively.

The DSSP-predicted secondary structures of proteins were encoded with a vocabulary of 12 tokens: m, H, B, E, G, I, P, T, S, K, Z, and -, corresponding to indices 0–11, respectively. The token m served as a mask (padding) token. The structural tokens of H, B, E, G, I, P, T, and S correspond to *α*-helix (H), residue in isolated *β*-bridge (B), extended strand participating in *β-*ladder (E), 310-helix (G), *π*-helix (I), *κ*-helix (P), hydrogen-bonded turn (T), bend (S), and none (-), respectively. The remaining tokens, K and Z, denote KRAB domain (K) and C2H2 ZF motif (Z), respectively. This encoding was processed by a transformer encoder module.

#### ProteinBERT architecture.

The ProteinBERT architecture (S1A Fig) comprises alternating global and local processing modules. The global module captures sequence-level contextual information, whereas the local module generates residue-specific embeddings. Cross-attention mechanisms facilitate bidirectional information exchange between these two modules, maintaining linear computational complexity with respect to sequence length, thereby ensuring scalability and efficiency for long protein sequences. The maximum protein length was set to 2,700 residues. Finally, a projection header was incorporated to align the embedding dimensionality with that required by downstream tasks.

#### Protein secondary structure encoder.

Protein secondary structural features (S1B Fig) were encoded using a transformer encoder applied iteratively. The resulting outputs were normalized via root-mean-square (RMS) normalization and subsequently served as the key and value embeddings in cross-attention with DNA embeddings.

#### COP architecture.

COP comprises five modules: a self-attention module for processing DNA embeddings, a cross-attention module that integrates DNA and protein embeddings, a cross-attention module that fuses DNA and secondary structure embeddings, a feedforward network (FFN), and a final classifier.

Input DNA sequences were first tokenized and projected into a 128-dimensional embedding space. These embeddings were then subjected to random dropout, followed by RMS normalization approach used in DeepSeek [81]. The normalized embeddings served as input to a self-attention module equipped with residual connections, enabling the model to capture long-range dependencies and global nucleotide interactions along the DNA sequence. Multi-head attention was implemented using torchtune [82], with rotary positional embeddings (RoPE) applied exclusively to the query and key projections, but not the value embeddings. Additionally, the torchtune implementation includes a linear output projection layer to transform the attention outputs into the desired feature dimension.

The output embedding of the DNA classification token was then RMS-normalized and passed to the downstream cross-attention module. Meanwhile, protein embeddings, extracted from the pretrained ProteinBERT model, were projected into key and value representations, allowing the model to capture contextual interactions between DNA and protein sequences. The resulting DNA-protein cross-attention output was subsequently RMS-normalized and fed into a second cross-attention layer. In this layer, embeddings encoding protein secondary structural features, including C2H2-ZF motifs, served as the key and value inputs, enabling the model to learn associations between protein secondary structure and DNA sequence.

The outputs underwent further processing by an FFN, which comprises an RMSNorm layer, followed by two linear transformations interleaved with a Gaussian error linear unit (GELU) activation function, and concluded with a dropout layer applying a stochastic mask. The entire pipeline, comprising self-attention, cross-attention, and FFN layers, was repeated multiple times. Finally, the embedding corresponding to the DNA classification token was normalized using RMSNorm and fed into the classifier head to produce the final logits.

This architecture encodes DNA sequence, protein sequence, and protein secondary structure representations independently in early stages, while enabling targeted, hierarchical information integration into the DNA representation via cascaded cross-attention. Transfer learning, leveraging the pretrained ProteinBERT weights, consistently yielded superior performance compared to de novo training.

#### Positive and negative samples.

For each of the 62 C2H2-ZFPs, 3,000 ChIP-seq binding sites were randomly sampled, yielding a total of 62 × 3,000 positive protein-DNA pairs. To generate negative pairs, each protein was paired with binding sites identified for the other 61 proteins, yielding 62 × 61 × 3,000 negative protein-DNA pairs. To address class imbalance, we employed hard negative sampling. Specifically, for each binding site in a batch of *B* positive pairs, the corresponding *B* × 61 negative pairs were filtered to exclude any that fell within a 300 bp window of the binding site. The remaining negatives were then ranked by their most recent loss values over previous training epochs. The top *R* (50%) highest-loss instances were selected as hard negatives, while the rest were sampled uniformly without replacement. This gradient-based one-sided sampling (GOSS) strategy [33] effectively preserved the overall negative distribution while adaptively enriching the training set with informative, challenging examples.

#### Benchmarking.

To evaluate the modeling performance (S6 Table), comparisons were made against both classical machine learning algorithms and a previously proposed deep learning method. For classical baselines, LightGBM and several standard classifiers, including support vector machine (with stochastic gradient descent learning), perceptron, passive-aggressive classifier, decision tree, random forest, AdaBoost, and naive Bayes, were implemented. To accommodate memory constraints, negative samples were downsampled to match the number of positive samples. For all baseline models, tokenized DNA, protein, and secondary structure sequences were concatenated as input features.

Ensemble-based methods, LightGBM and AdaBoost (boosting) as well as Random Forest (bagging), achieved the highest accuracies, approximately 0.59. Notably, naive Bayes outperformed linear models of SVM, perceptron, and passive-aggressive classifier, suggesting that C2H2-ZFP-DNA recognition has inherent non-linear dependencies. The deep learning model DeepZF [26] was also benchmarked. DeepZF comprises two components. One is classification of individual C2H2 ZF into DNA-binding versus non-binding categories. The other is prediction of triple-nucleotide motif for a C2H2 ZF. For each protein, the predicted triple-nucleotide motifs from tandem C2H2 fingers were concatenated to generate a composite DNA binding motif. These composite motifs were then scored against positive and negative DNA binding sites using FIMO (Find Individual Motif Occurrences). A score threshold was optimized on 90% of the ChIP-seq dataset and evaluated on the remaining 10%. As shown in S7 Table, DeepZF did not outperform the ensemble learning methods or other common classifiers in predicting binding sites.

### Bioinformatics analyses

#### Data analyses of RNA-seq.

RNA-seq raw FASTQ files were aligned to the mouse reference genome (GRCm38) using Hisat2, generating Sequence Alignment Map (SAM) files. These SAM files were converted into sorted, indexed Binary Alignment Map (BAM) files using Samtools (v1.15.1). The generated BAM files were processed using Cufflinks to quantify transcript expression levels in units of fragments per kilobase of variable exon per million fragments mapped (FPKM). Raw read counts were subsequently imported into DESeq2 for differential gene expression analyses, with significantly differentially expressed genes defined as those exhibiting an absolute log_2_ fold change > 0.5 and an adjusted *p*-value < 0.05.

Manhattan plots depicting localized enrichments of downregulated genes were generated using the CMplot package (v4.5.1) in R. The mouse reference genome was partitioned into non-overlapping 1-Mb bins. The genomic locations of downregulated genes were intersected with these bins to calculate the frequency of occurrences within each bin. The distribution of occurrences was modeled using a Poisson distribution, with the maximum-likelihood estimator of λ derived from the mean frequency of events. Probabilities of observing the signals in each bin were then computed under the Poisson model and visualized with CMplot to produce Manhattan plots.

#### Data analyses of ChIP-seq.

Raw reads were trimmed using fastp (v0.21.0) to remove the ﬁrst 10-bp barcode and adaptor sequences. Trimmed reads were then aligned to the mouse reference genome (mm9) using Bowtie2 (v2.3.4.1), generating SAM files. These SAM ﬁles were converted, sorted, and indexed into BAM files using Samtools (v1.15.1). For comparative analysis of protein-DNA occupancy across samples, BAM files were normalized to reads per kilobase per million mapped reads (RPKM) with a bin size of 20 bp using the bamCoverage module in deepTools. The resulting bedGraph ﬁles were uploaded to the UCSC Genome Browser for visualization of genomic regions of interest. Narrow peaks were identified using MACS (v1.4.2 20120305) with input controls as the background and a stringent *q*-value cutoff of 0.001. Heatmaps depicting signal distribution were generated using the plotHeatmap module of deepTools. To assess enrichment at each *cPcdh* gene, the peak summit within the corresponding pCBS element was identified, and its signal intensity was used as the enrichment score. For motif analyses, the MEME suite (v4.12.0) and HOMER (v5.1) were used for DNA motif analyses.

#### Data analyses of QHR-4C.

For the QHR-4C data, P7 reads were demultiplexed based on unique barcode-index combinations. Anchor primer sequences were trimmed, and PCR duplicates were removed using FastUniq (v1.1). The resulting non-redundant reads were aligned to the mouse reference genome (mm9) using Bowtie2 (v2.3.5) to generate SAM files, which were then converted to sorted, indexed BAM files. BAM files were processed with the r3Cseq package (v1.20) in R (v3.3.3) to compute normalized read counts as reads per million (RPM). BedGraph files generated by r3Cseq were uploaded to the UCSC Genome Browser for visualization. Loop intensities were quantified using the RPKM.

#### Quantification and statistics.

RNA-seq, ChIP-seq, and QHR-4C experiments were performed with at least two biological replicates. All statistical tests were calculated using GraphPad (v8.4.2), R (v4.3.3) or Python (v3.6.9) scripts. Data were presented as mean ± standard deviation (SD). The values of statistical signiﬁcance were calculated using an unpaired Student’s t-test, with significance levels denoted as follows: ‘*’ for p ≤ 0.05, ‘**’ for p ≤ 0.01, ‘***’ for p ≤ 0.001, and ‘****’ for p ≤ 0.0001.

## Supporting information

S1 Fig Model architectures and predicted zinc finger distribution. **(A)** Architecture of the ProteinBERT model for deep learning on protein sequences. Protein residue-wise features are processed by narrow and wide convolutions. Cross-attention operates between a single global embedding and multiple residue embeddings, resulting in linear complexity with protein length. Bidirectional information flow between local and global representations allows residues to depend on each other. A projection head ensures output dimensionality consistent with COP. GELU, Gaussian error linear unit. **(B)** Architecture of the protein secondary structure encoder, implemented as a standard transformer. RMS normalization was used in place of layer normalization and applied prior to the attention module. **(C-L)** COP predicts 34 C2H2-ZFP members potentially occupy all 54 *cPcdh* promoter CBS elements, of which 5 contain single C2H2 zinc finger (ZF) domain **(C)**, 1 contains two C2H2-ZFs **(D)**, 7 contain three C2H2-ZFs **(E)**, 2 contain four C2H2-ZFs **(F)**, 8 contain five C2H2-ZFs **(G)**, 6 contain six C2H2-ZFs **(H)**, 2 contain seven C2H2-ZFs **(I)**, 1 contains nine C2H2-ZFs **(J)**, 1 contains ten C2H2-ZFs **(K)**, and 1 contains twelve C2H2-ZFs **(L)**. Blue box indicates C2H2-ZF domain.
(TIF)

S2 Fig COP prediction of C2H2-ZFP occupancy at the *cPcdh* pCBS elements. **(A-P)** COP-predicted occupancy ratio at the 54 *cPcdh* pCBS elements for each C2H2-ZFP containing single C2H2-ZF domain **(A)**, as well as containing 2 **(B)**, 3 **(C)**, 4 **(D)**, 5 **(E)**, 6 **(F)**, 7 **(G)**, 8 **(H)**, 9 **(I)**, 10 **(J)**, 11 **(K)**, 12 **(L)**, 13 **(M)**, 14 **(N)**, 15 **(O)**, or 16–30 **(P)** C2H2-ZFs. C2H2-ZFPs are colored in green for trained or black for untrained. The protein numbers for each ZFP group with 1–30 ZFs are indicated in parentheses.
(TIF)

S3 Fig Generation of N2a single-cell clones with *Wiz* Myc-tagged or deleted. **(A)** Generation of N2a single-cell clones with endogenous *Wiz* Myc-tagged at the C-terminus. A Myc-coding sequence was inserted immediately upstream of the stop codon (TAA) of the endogenous *Wiz* gene via single-sgRNA-guided Cas9 cleavage followed by DNA template-mediated homology-directed repair (HDR). C-terminal Myc tagging was confirmed in single-cell clone by genotyping with Sanger sequencing. The inserted Myc tag is indicated in orange. **(B)** Generation of *Wiz*-knockout N2a single-cell clones via CRISPR genome editing. Two sgRNAs were designed to program Cas9 cleavage within introns 2 and 13 of the *Wiz* gene, respectively, enabling excision of the intervening genomic fragment. Genotyping of *Wiz*-knockout (Δ*Wiz*) single-cell clones by Sanger sequencing confirmed large, targeted deletions. Junction sequence analyses revealed substantial allelic heterogeneity, attributable to variable end resection and error-prone non-homologous end joining (NHEJ) repair at the double-strand breaks (DSBs) on individual alleles.
(TIF)

S4 Fig Loss of *Wiz* increases *cPcdh* expression levels in N2a cells. **(A-C)** RNA-seq profiles **(A)** and quantification **(B)**, as well as Western blot **(C)** confirming Wiz deletion in Δ*Wiz* N2a single-cell clones. **(D)** Volcano plot depicting differentially expressed genes in neuronal N2a cells upon *Wiz* knockout. Red, upregulated (log_2_ fold change (FC) > 0.5, p < 0.05); Blue, downregulated (log_2_ FC < -0.5, p < 0.05); Gray, not significant. Dotted square, enlarged in Fig 4A. **(E)** Association between Wiz ChIP-seq peak density near transcription start sites (TSS; ± 2 kb) and expression changes induced by Wiz loss (RNA-seq) in N2a cells. **(F)** Heatmaps showing increased RNA-seq expression levels of *cPcdh* in N2a cells upon *Wiz* deletion. **(G)** Bar plots depicting fold changes of gene expression levels of *cPcdh* in N2a cells upon *Wiz* deletion. RNA-seq was performed in duplicate for each cell sample. For Δ*Wiz* N2a cells, data from two independent clones were merged. FPKM, fragments per kilobase of exon per million reads mapped. Data as mean ± standard deviation (SD); Unpaired Student’s *t*-test. ****p ≤ 0.0001.
(TIF)

S5 Fig Increased enrichments of active chromatin marks of H3K4me3 and H3K27ac at *cPcdh* regulatory elements in N2a cells upon *Wiz* deletion. **(A-F)** ChIP-seq profiles of H3K4me3 and H3K27ac at the *Pcdh α* (**A** and **B)**, *β* (**C** and **D)**, and *γ* (**E** and **F)** gene clusters in ∆*Wiz* N2a single-cell clones compared to wild-type (WT) control clone. **(G)** Violin plots of ChIP-seq signals at all promoter and enhancer regions of the *cPcdh* locus reveal increased enrichments of both H3K4me3 and H3K27ac, marks of active chromatin, upon *Wiz* deletion. ChIP-seq signals were normalized using RPKM (reads per kilobase per million mapped reads). For WT N2a cells, H3K4me3 and H3K27ac signals were merged from two and three replicates, respectively. For Δ*Wiz* N2a cells, data from two independent knockout clones were combined, each with two (H3K4me3) or three (H3K27ac) replicates.
(TIF)

S6 Fig Generation of *Wiz* conditional knockout mice via pronuclei injection. **(A)** Schematic of CRISPR/Cas9-mediated homologous recombination (HR) for generating conditional *Wiz* knockout mouse model. Two *loxP* sites were inserted into introns 5 and 13 of the *Wiz* gene via Cas9-induced double-strand breaks (DSBs), guided by single sgRNAs and repaired using donor DNA templates with the loxP sites. **(B)** Genotyping of the homologous *Wiz*-floxed (*Wiz*^f/f^) mouse strain by Sanger sequencing confirming targeted insertion of *loxP* sites flanking exons 6–13 of the *Wiz* gene.
(TIF)

S7 Fig Loss of *Wiz* increases *cPcdh* expression levels in mouse cortices. **(A-C)** RNA-seq profiles **(A)** and quantification **(B)**, as well as Western blot **(C)** confirming *Wiz* deletion in the postnatal day 0 (P0) mouse cortices of conditional *Wiz* knockout (*Wiz*^cKO^) versus the *Wiz*^f/f^ control mice. *Wiz*^f/f^ represents *Wi*z^f/f^;*Emx1-Cre*^-^ mice; *Wiz*^cKO^ represents *Wiz*^f/f^;*Emx1-Cre*^+^ mice. **(D)** Volcano plot depicting differentially expressed genes in P0 mouse cortices upon conditional knockout of *Wiz*. Red: upregulated (log_2_ fold change (FC) > 0.5, p < 0.05); Blue: downregulated (log_2_ FC < -0.5, p < 0.05); Gray: not significant. The enlarged region is shown in Fig 4F. **(E)** Heatmaps showing increased *cPcdh* expression levels in mouse cortices upon *Wiz* knockout *in vivo*. **(F)** Bar plots depicting fold changes of gene expression levels of *cPcdh* in mouse cortices upon *Wiz* knockout. For each genotype (*Wiz*^f/f^ and *Wiz*^cKO^), two individual mice were used and the data from two biological replicates were merged. FPKM, fragments per kilobase of exon per million reads mapped. Data as mean ± standard deviation (SD); Unpaired Student’s *t*-test. ****p ≤ 0.0001.
(TIF)

S8 Fig Repressive chromatin marks of H3K9 mono-, bi-, and tri-methylations show no obvious alteration in N2a cells upon *Wiz* deletion. **(A-C)** ChIP-seq profiles showing no obvious alterations of repressive chromatin marks of H3K9me1, H3K9me2, and H3K9me3 across *Pcdh α* **(A)**, *β* **(B)**, and *γ* **(C)** clusters in N2a cells. ChIP-seq signals were normalized using RPKM (reads per kilobase per million mapped reads). For WT N2a cells, H3K9me1, H3K9me2, and H3K9me3 signals were merged from four, five, and four replicates, respectively. For Δ*Wiz* N2a cells, data from two independent deletion clones were combined, each with four (H3K9me1), five (H3K9me2), four (H3K9me3) replicates.
(TIF)

S9 Fig Effect of Wiz loss on H3K9 methylations and cohesin positioning at CTCF sites in N2a cells. (**A-C**) Quantification of H3K9me1 **(A)**, H3K9me2 **(B)**, and H3K9me3 **(C)** ChIP-seq signals at the promoters of the *Pcdh* clusters in Δ*Wiz* compared to WT N2a cells. **(D-F)** Global ChIP-seq profiles across gene bodies in Δ*Wiz* compared to WT N2a cells, showing no significant differences at transcription start sites (TSSs) for H3K9me1 **(D)**, H3K9me2 **(E)**, and H3K9me3 **(F)** upon *Wiz* deletion. **(G)** Distribution of Wiz and Rad21 ChIP-seq peak summits relative to the forward-oriented CTCF sites in N2a cells. **(H)** Distribution of Rad21 ChIP-seq peak summits in ∆*Wiz* compared to WT cells, showing a ~ 3 bp shift of cohesin toward CTCF sites upon *Wiz* deletion in N2a cells. **(I)** A “molecular brake” model for Wiz. Wiz co-occupancy with CTCF fine-tunes cohesin sliding, restricting its processivity and extrusion distance. Loss of *Wiz* leads to aberrant long-range enhancer-promoter interactions.
(TIF)

S1 Table Datasets used in this study. (XLSX)

S2 Table Differentially expressed genes (DEGs) in Δ*Wiz* compared to WT N2a cells. (XLSX)

S3 Table Differentially expressed genes (DEGs) in *Wiz*^cKO^ compared to *Wiz*^f/f^ mouse cortices. (XLSX)

S4 Table Oligonucleotides used in this study. (XLSX)

S5 Table ChIP-seq datasets generated in this study. (XLSX)

S6 Table COP prediction of C2H2-ZFPs’ occupancy at the pCBS elements of the *Pcdh* clusters. (XLSX)

S7 Table Performance comparison of COP and common machine learning classifiers. (XLSX)

S1 Data Source Data for all data presented in the figures are listed in the file. Fig 4B: Table F4B in S1 Data. Fig 4C-4E: Table F4CDE in S1 Data. Fig 4G: Table F4G in S1 Data. Fig 4H-4J: Table F4HIJ in S1 Data. Fig 5I and 5J: Table F5IJ in S1 Data. Fig 6A and 6B: Table F6AB in S1 Data. Fig 6C and 6D: Table F6CD in S1 Data. Fig 6F-6H: Table F6FGH in S1 Data. S4E Fig: Table S4E in S1 Data. S5G Fig: Table S5G in S1 Data. S9A-S9C Fig: Table S9ABC in S1 Data.
(XLSX)

S1 Raw Images Uncropped original Western blot images for S4C and S7C Figs. (PDF)
