# Supplementary figures and images for: Wiz regulates clustered protocadherin genes by restricting CTCF/cohesin loop extrusion in a genomic-distance biased manner
Source: PLoS Genet. 2026 Jul 16;22(7):e1012242. doi: 10.1371/journal.pgen.1012242 (PMC13395409; doi:10.1371/journal.pgen.1012242)

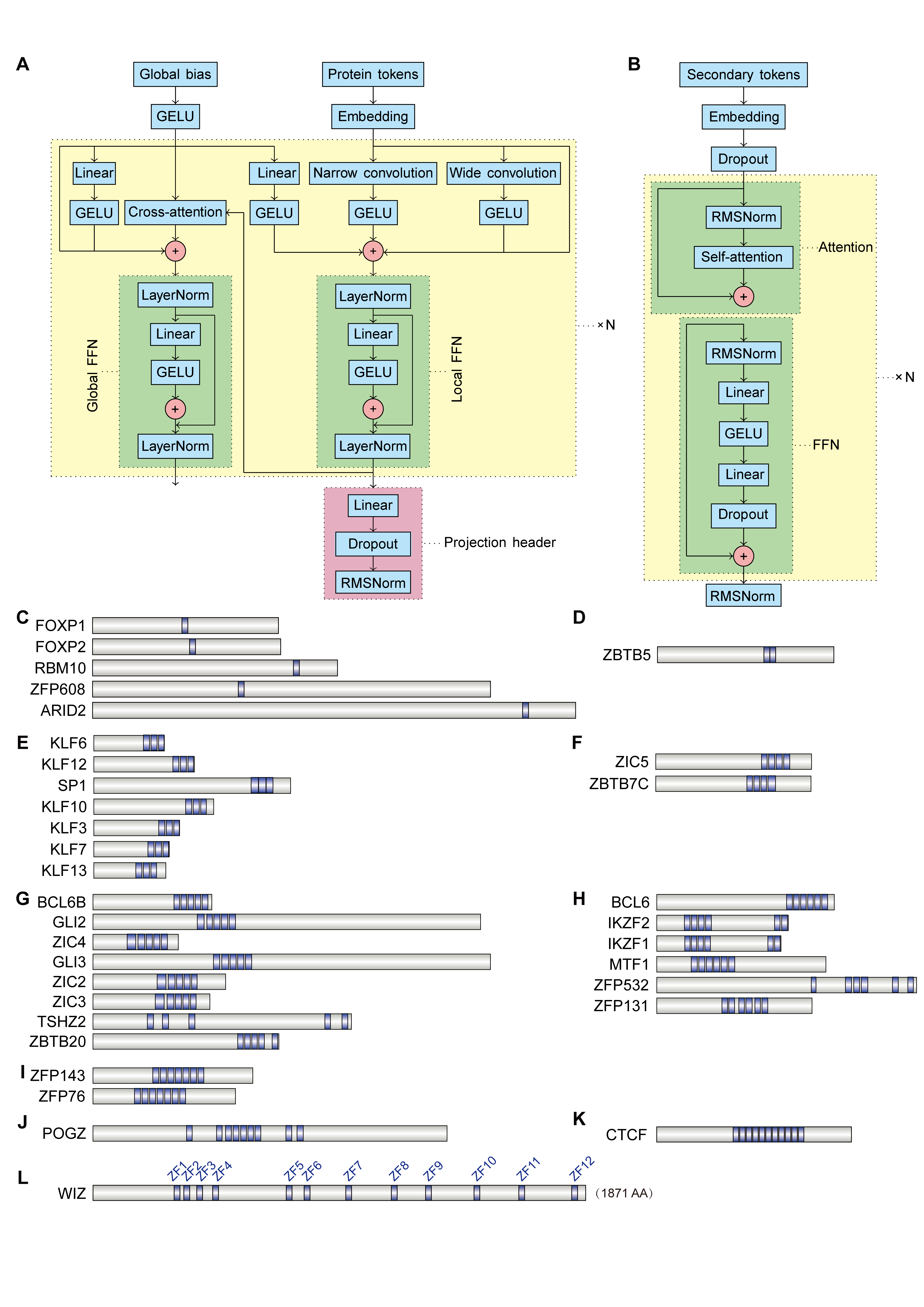

Supplement: S1 Fig — (A) Architecture of the ProteinBERT model for deep learning on protein sequences. Protein residue-wise features are processed by narrow and wide convolutions. Cross-attention operates between a single global embedding and multiple residue embeddings, resulting in linear complexity with protein length. Bidirectional information flow between local and global representations allows residues to depend on each other. A projection head ensures output dimensionality consistent with COP. GELU, Gaussian error linear unit. (B) Architecture of the protein secondary structure encoder, implemented as a standard transformer. RMS normalization was used in place of layer normalization and applied prior to the attention module. (C-L) COP predicts 34 C2H2-ZFP members potentially occupy all 54 cPcdh promoter CBS elements, of which 5 contain single C2H2 zinc finger (ZF) domain (C), 1 contains two C2H2-ZFs (D), 7 contain three C2H2-ZFs (E), 2 contain four C2H2-ZFs (F), 8 contain five C2H2-ZFs (G), 6 contain six C2H2-ZFs (H), 2 contain seven C2H2-ZFs (I), 1 contains nine C2H2-ZFs (J), 1 contains ten C2H2-ZFs (K), and 1 contains twelve C2H2-ZFs (L). Blue box indicates C2H2-ZF domain. (TIF) [file pgen.1012242.s001.tif]

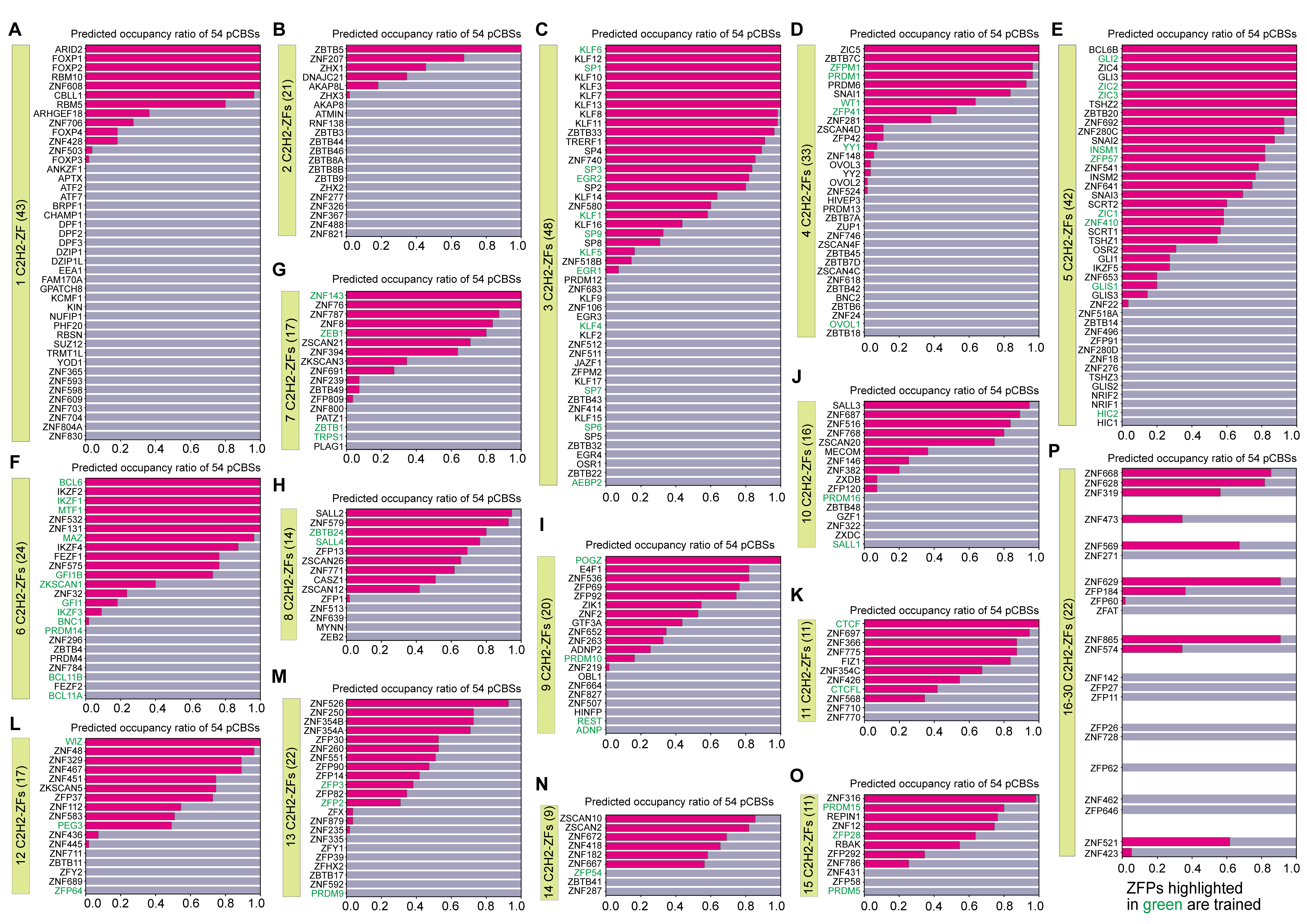

Supplement: S2 Fig — (A-P) COP-predicted occupancy ratio at the 54 cPcdh pCBS elements for each C2H2-ZFP containing single C2H2-ZF domain (A), as well as containing 2 (B), 3 (C), 4 (D), 5 (E), 6 (F), 7 (G), 8 (H), 9 (I), 10 (J), 11 (K), 12 (L), 13 (M), 14 (N), 15 (O), or 16–30 (P) C2H2-ZFs. C2H2-ZFPs are colored in green for trained or black for untrained. The protein numbers for each ZFP group with 1–30 ZFs are indicated in parentheses. (TIF) [file pgen.1012242.s002.tif]

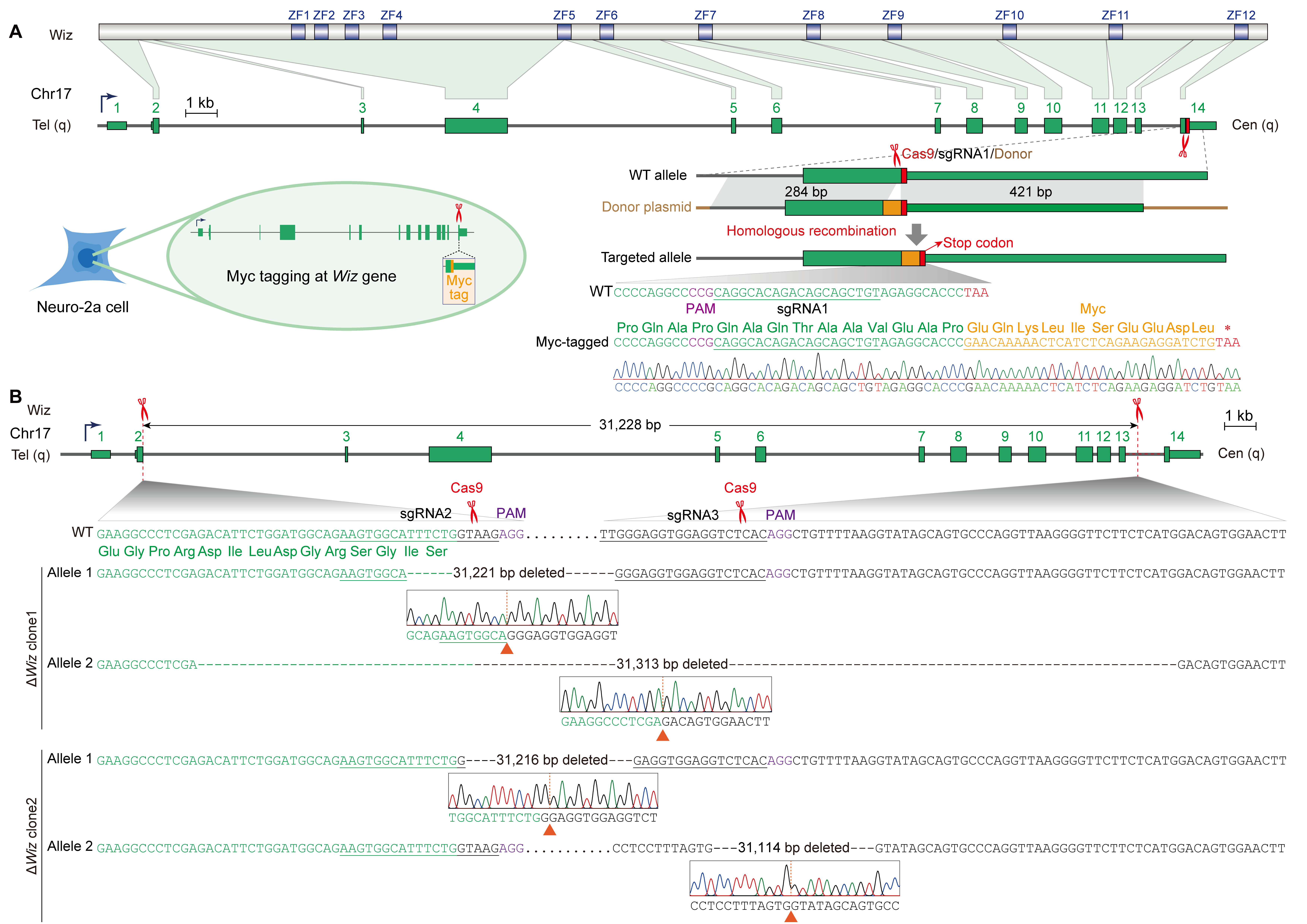

Supplement: S3 Fig — (A) Generation of N2a single-cell clones with endogenous Wiz Myc-tagged at the C-terminus. A Myc-coding sequence was inserted immediately upstream of the stop codon (TAA) of the endogenous Wiz gene via single-sgRNA-guided Cas9 cleavage followed by DNA template-mediated homology-directed repair (HDR). C-terminal Myc tagging was confirmed in single-cell clone by genotyping with Sanger sequencing. The inserted Myc tag is indicated in orange. (B) Generation of Wiz-knockout N2a single-cell clones via CRISPR genome editing. Two sgRNAs were designed to program Cas9 cleavage within introns 2 and 13 of the Wiz gene, respectively, enabling excision of the intervening genomic fragment. Genotyping of Wiz-knockout (ΔWiz) single-cell clones by Sanger sequencing confirmed large, targeted deletions. Junction sequence analyses revealed substantial allelic heterogeneity, attributable to variable end resection and error-prone non-homologous end joining (NHEJ) repair at the double-strand breaks (DSBs) on individual alleles. (TIF) [file pgen.1012242.s003.tif]

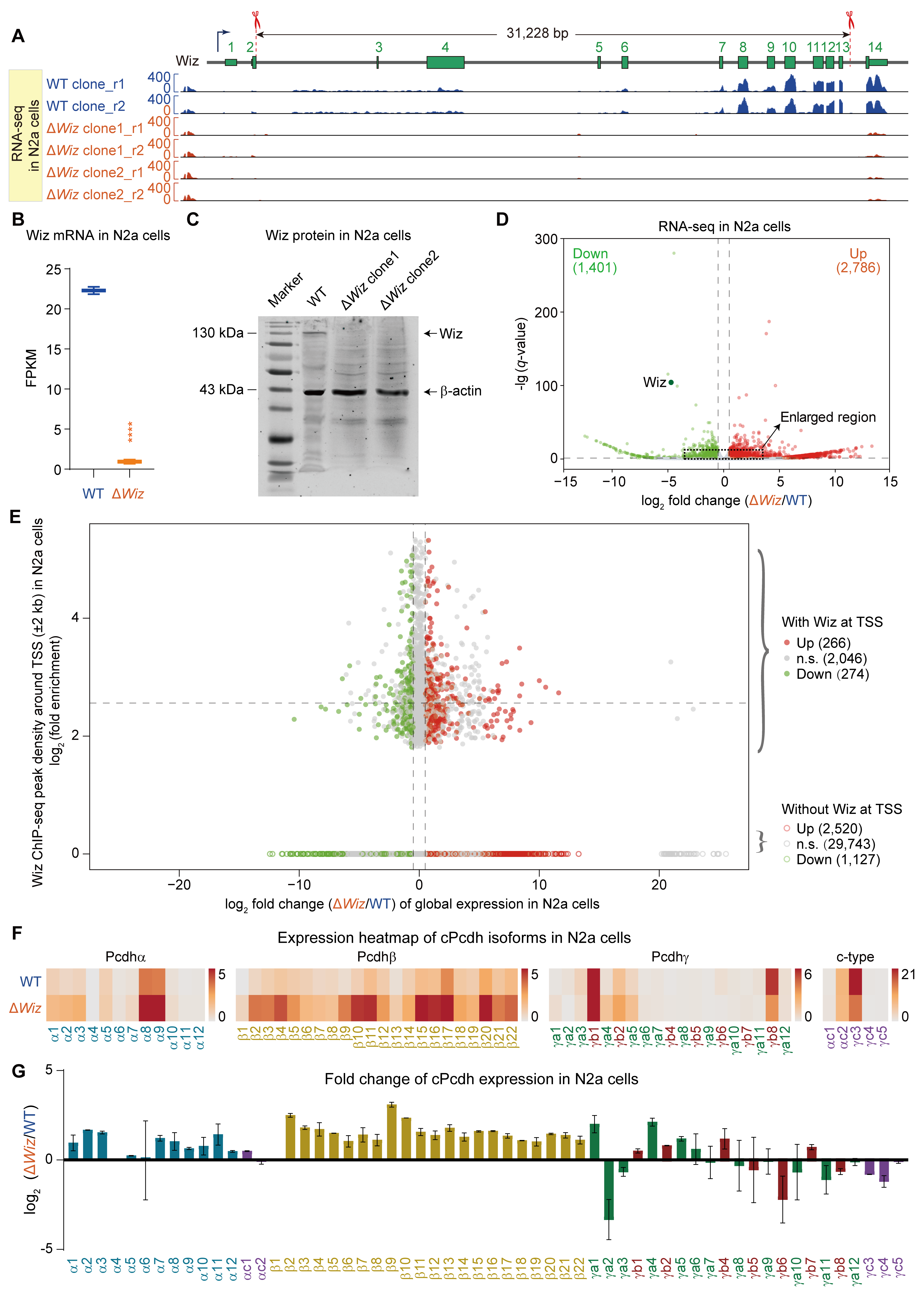

Supplement: S4 Fig — (A-C) RNA-seq profiles (A) and quantification (B), as well as Western blot (C) confirming Wiz deletion in ΔWiz N2a single-cell clones. (D) Volcano plot depicting differentially expressed genes in neuronal N2a cells upon Wiz knockout. Red, upregulated (log2 fold change (FC) > 0.5, p < 0.05); Blue, downregulated (log2 FC < -0.5, p < 0.05); Gray, not significant. Dotted square, enlarged in Fig 4A. (E) Association between Wiz ChIP-seq peak density near transcription start sites (TSS; ± 2 kb) and expression changes induced by Wiz loss (RNA-seq) in N2a cells. (F) Heatmaps showing increased RNA-seq expression levels of cPcdh in N2a cells upon Wiz deletion. (G) Bar plots depicting fold changes of gene expression levels of cPcdh in N2a cells upon Wiz deletion. RNA-seq was performed in duplicate for each cell sample. For ΔWiz N2a cells, data from two independent clones were merged. FPKM, fragments per kilobase of exon per million reads mapped. Data as mean ± standard deviation (SD); Unpaired Student’s t-test. ****p ≤ 0.0001. (TIF) [file pgen.1012242.s004.tif]

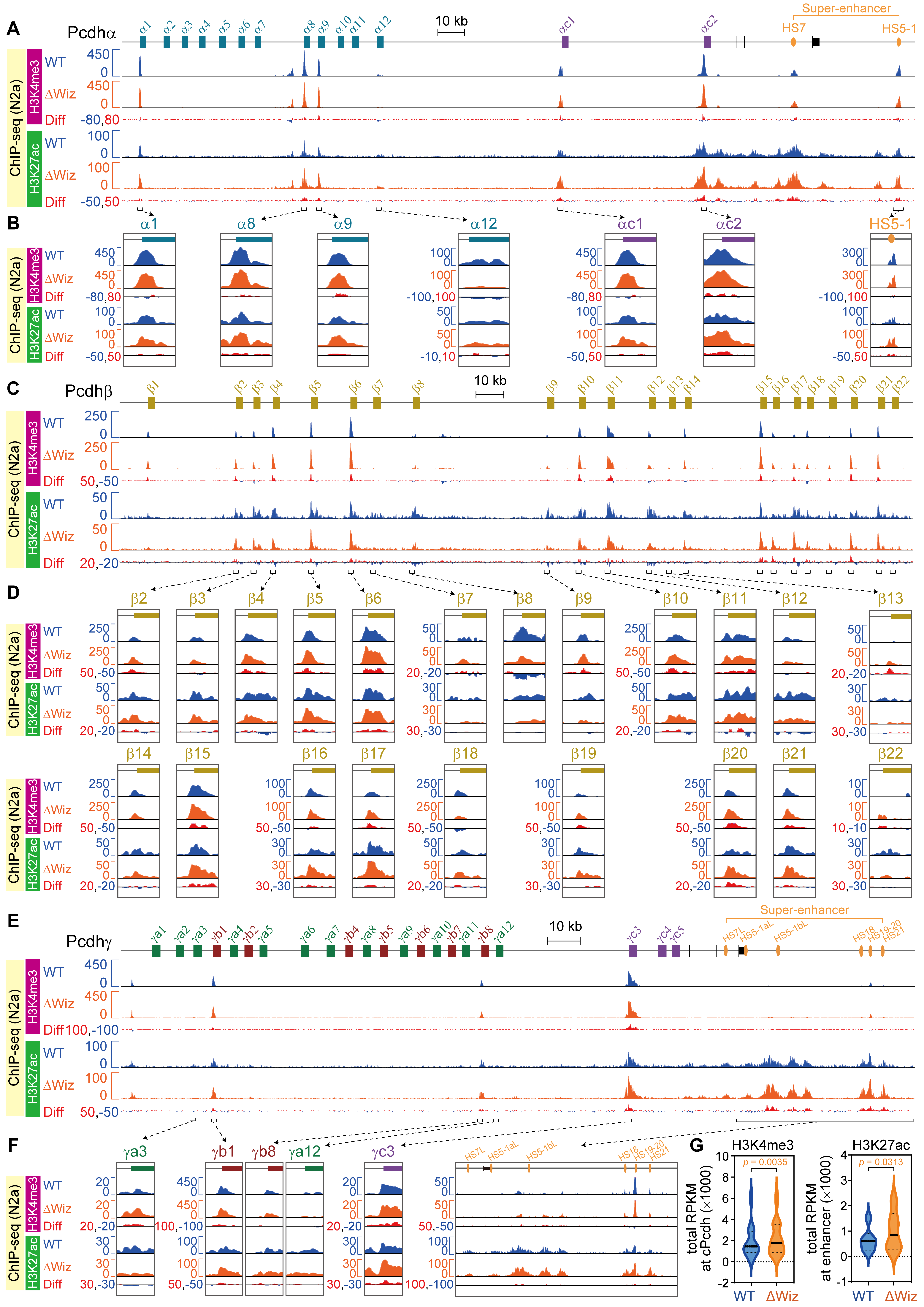

Supplement: S5 Fig — (A-F) ChIP-seq profiles of H3K4me3 and H3K27ac at the Pcdh α (A and B), β (C and D), and γ (E and F) gene clusters in ∆Wiz N2a single-cell clones compared to wild-type (WT) control clone. (G) Violin plots of ChIP-seq signals at all promoter and enhancer regions of the cPcdh locus reveal increased enrichments of both H3K4me3 and H3K27ac, marks of active chromatin, upon Wiz deletion. ChIP-seq signals were normalized using RPKM (reads per kilobase per million mapped reads). For WT N2a cells, H3K4me3 and H3K27ac signals were merged from two and three replicates, respectively. For ΔWiz N2a cells, data from two independent knockout clones were combined, each with two (H3K4me3) or three (H3K27ac) replicates. (TIF) [file pgen.1012242.s005.tif]

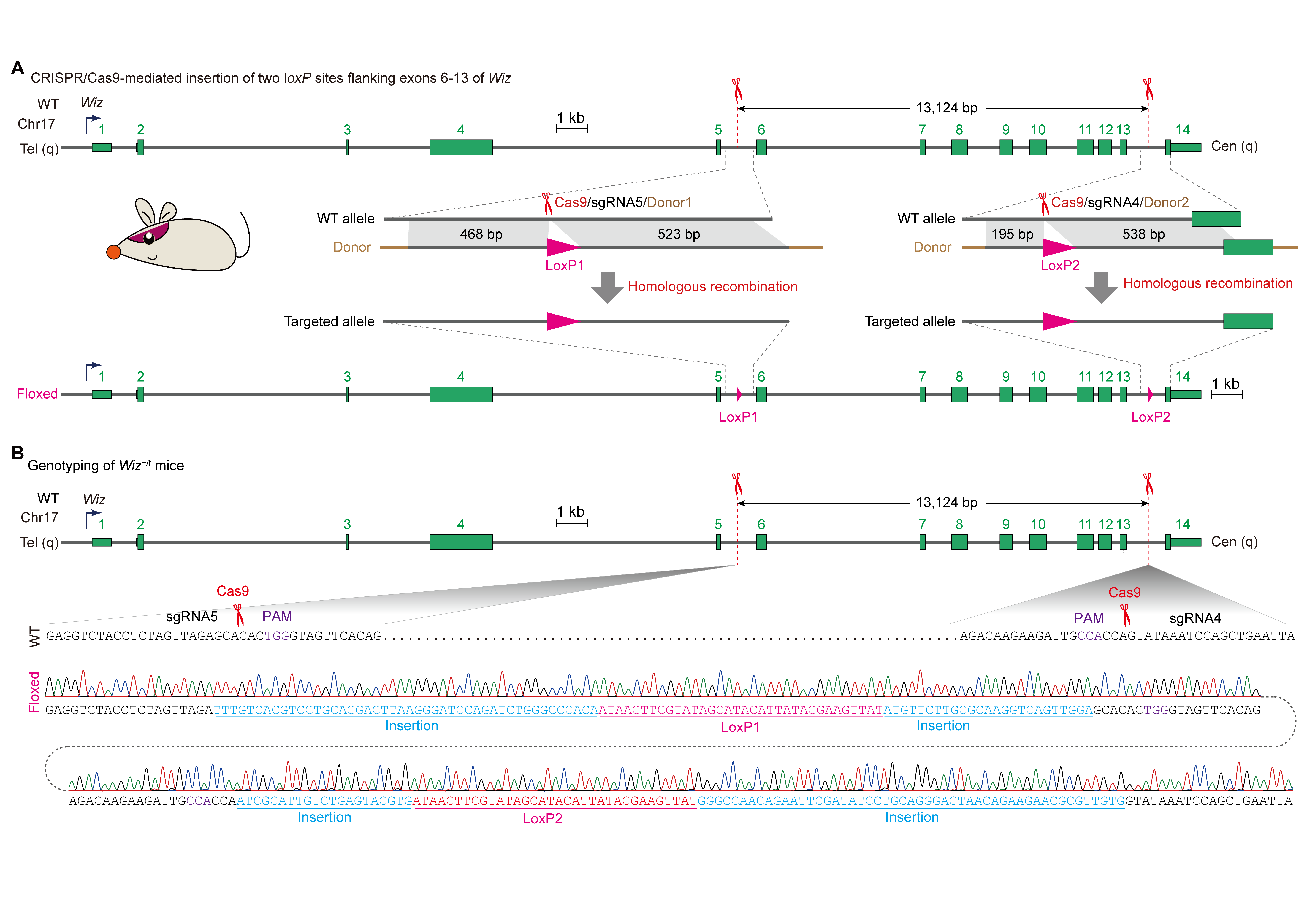

Supplement: S6 Fig — (A) Schematic of CRISPR/Cas9-mediated homologous recombination (HR) for generating conditional Wiz knockout mouse model. Two loxP sites were inserted into introns 5 and 13 of the Wiz gene via Cas9-induced double-strand breaks (DSBs), guided by single sgRNAs and repaired using donor DNA templates with the loxP sites. (B) Genotyping of the homologous Wiz-floxed (Wizf/f) mouse strain by Sanger sequencing confirming targeted insertion of loxP sites flanking exons 6–13 of the Wiz gene. (TIF) [file pgen.1012242.s006.tif]

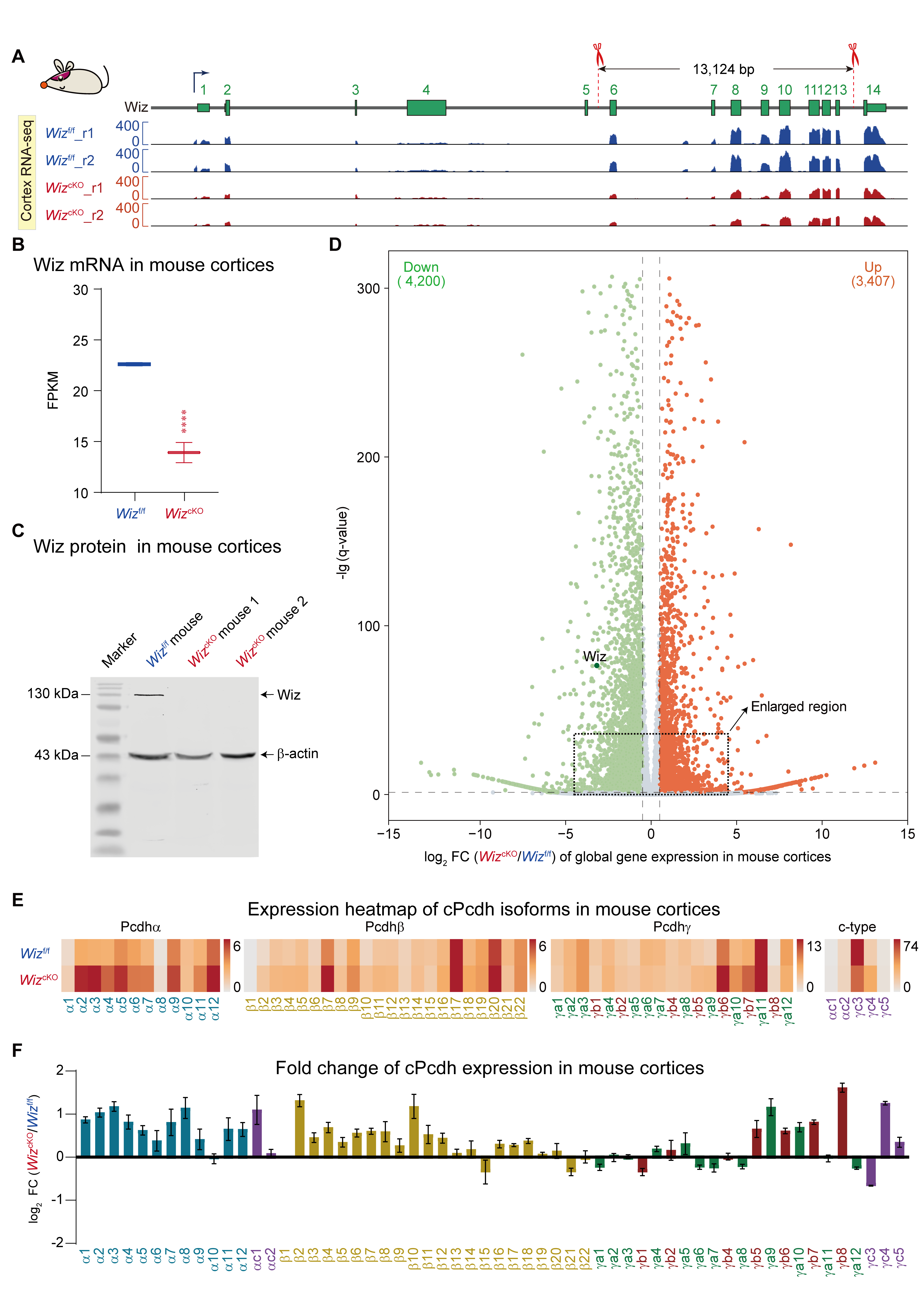

Supplement: S7 Fig — (A-C) RNA-seq profiles (A) and quantification (B), as well as Western blot (C) confirming Wiz deletion in the postnatal day 0 (P0) mouse cortices of conditional Wiz knockout (WizcKO) versus the Wizf/f control mice. Wizf/f represents Wizf/f;Emx1-Cre- mice; WizcKO represents Wizf/f;Emx1-Cre+ mice. (D) Volcano plot depicting differentially expressed genes in P0 mouse cortices upon conditional knockout of Wiz. Red: upregulated (log2 fold change (FC) > 0.5, p < 0.05); Blue: downregulated (log2 FC < -0.5, p < 0.05); Gray: not significant. The enlarged region is shown in Fig 4F. (E) Heatmaps showing increased cPcdh expression levels in mouse cortices upon Wiz knockout in vivo. (F) Bar plots depicting fold changes of gene expression levels of cPcdh in mouse cortices upon Wiz knockout. For each genotype (Wizf/f and WizcKO), two individual mice were used and the data from two biological replicates were merged. FPKM, fragments per kilobase of exon per million reads mapped. Data as mean ± standard deviation (SD); Unpaired Student’s t-test. ****p ≤ 0.0001. (TIF) [file pgen.1012242.s007.tif]

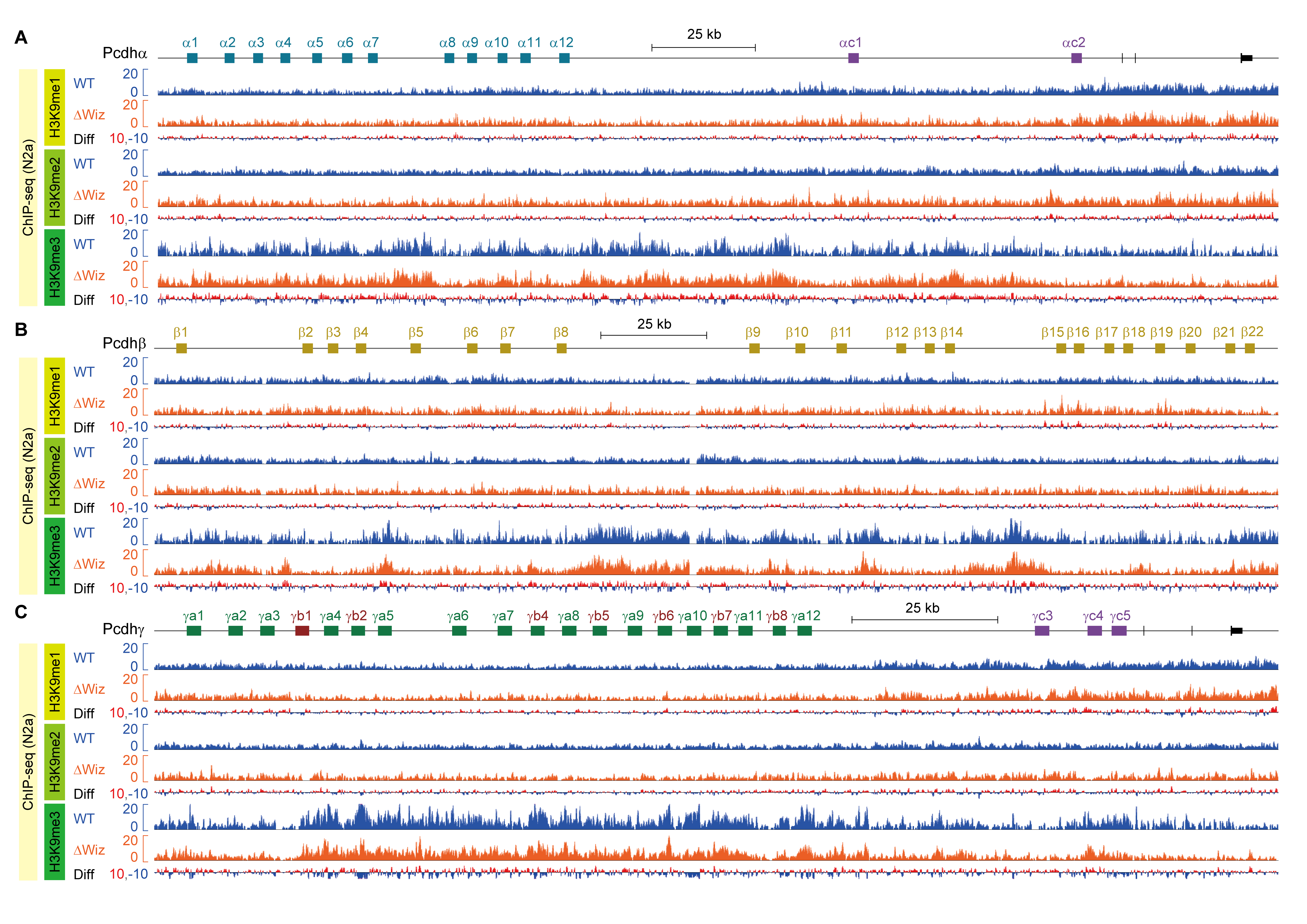

Supplement: S8 Fig — (A-C) ChIP-seq profiles showing no obvious alterations of repressive chromatin marks of H3K9me1, H3K9me2, and H3K9me3 across Pcdh α (A), β (B), and γ (C) clusters in N2a cells. ChIP-seq signals were normalized using RPKM (reads per kilobase per million mapped reads). For WT N2a cells, H3K9me1, H3K9me2, and H3K9me3 signals were merged from four, five, and four replicates, respectively. For ΔWiz N2a cells, data from two independent deletion clones were combined, each with four (H3K9me1), five (H3K9me2), four (H3K9me3) replicates. (TIF) [file pgen.1012242.s008.tif]

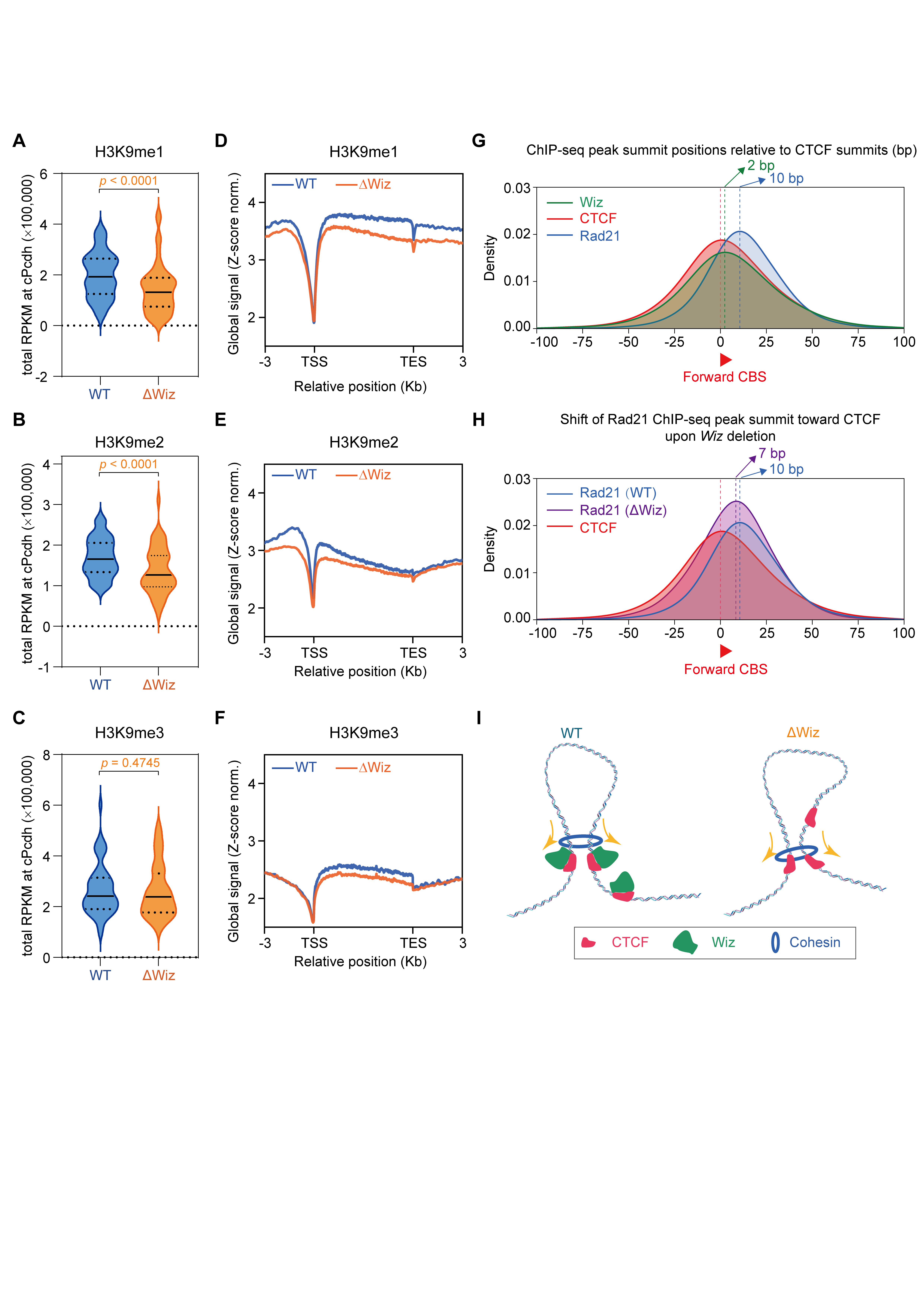

Supplement: S9 Fig — (A-C) Quantification of H3K9me1 (A), H3K9me2 (B), and H3K9me3 (C) ChIP-seq signals at the promoters of the Pcdh clusters in ΔWiz compared to WT N2a cells. (D-F) Global ChIP-seq profiles across gene bodies in ΔWiz compared to WT N2a cells, showing no significant differences at transcription start sites (TSSs) for H3K9me1 (D), H3K9me2 (E), and H3K9me3 (F) upon Wiz deletion. (G) Distribution of Wiz and Rad21 ChIP-seq peak summits relative to the forward-oriented CTCF sites in N2a cells. (H) Distribution of Rad21 ChIP-seq peak summits in ∆Wiz compared to WT cells, showing a ~ 3 bp shift of cohesin toward CTCF sites upon Wiz deletion in N2a cells. (I) A “molecular brake” model for Wiz. Wiz co-occupancy with CTCF fine-tunes cohesin sliding, restricting its processivity and extrusion distance. Loss of Wiz leads to aberrant long-range enhancer-promoter interactions. (TIF) [file pgen.1012242.s009.tif]
