# Supplementary material for: Wiz regulates clustered protocadherin genes by restricting CTCF/cohesin loop extrusion in a genomic-distance biased manner
Source: PLoS Genet. 2026 Jul 16;22(7):e1012242. doi: 10.1371/journal.pgen.1012242 (PMC13395409; doi:10.1371/journal.pgen.1012242)

A

Western blotting with an anti-Wiz antibody
in Δ*Wiz* versus WT N2a cell clones

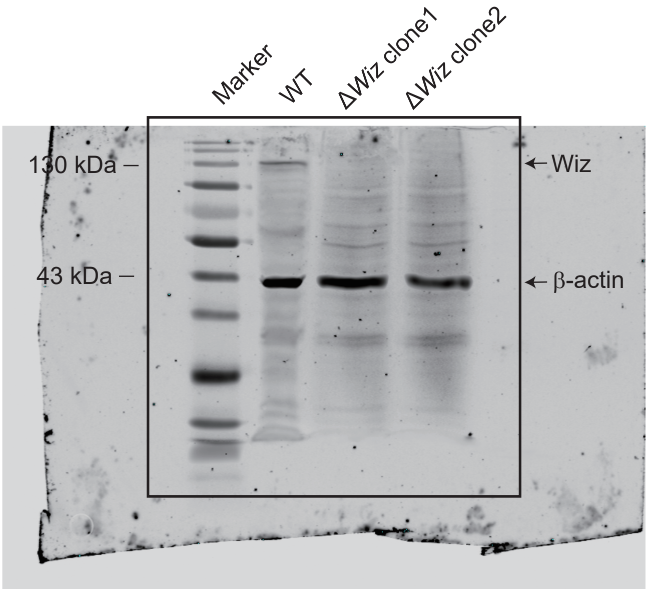

B

Western blotting with an anti-Wiz antibody
in P0 cotices of *Wiz*^{cKO} versus the *Wiz*$^{f/f}$ mice

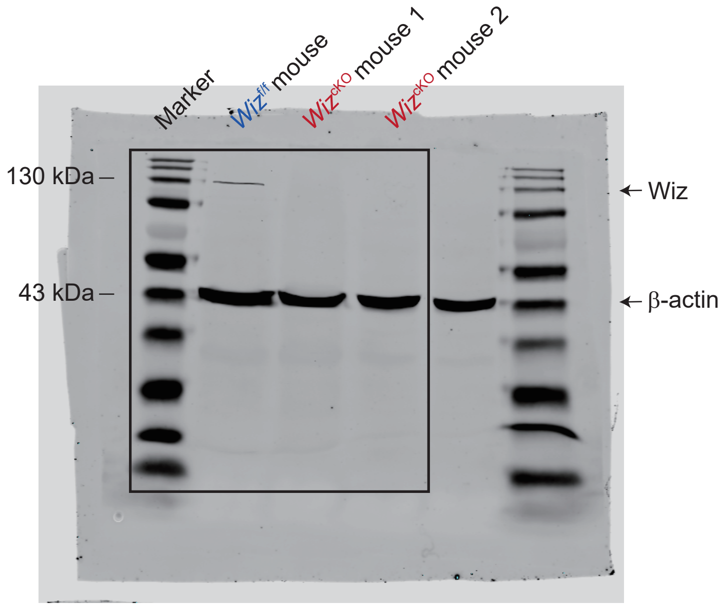

Supplement: S1 Raw Images — (PDF) [file pgen.1012242.s018.pdf]
